# A Hemoglobin-Based Nanoparticle Delivery System Enhances the Pharmacokinetics and Efficacy of Tigecycline in *Klebsiella pneumoniae* Infections

**DOI:** 10.34133/bmef.0241

**Published:** 2026-03-30

**Authors:** Xingfang Qiao, Yiwen Hao, Yinglan Yu, Zhe Qiang, Xingjian Wen, Yanlei Guo, Zhen Wu, Hao Shao, Lei Luo

**Affiliations:** ^1^ College of Pharmaceutical Sciences, Southwest University, Chongqing 400715, China.; ^2^ Chongqing Academy of Chinese Materia Medica, Chongqing 400065, China.

## Abstract

**Objective:** This study aims to engineer a tigecycline–hemoglobin nanoparticle (TIG–HBNP) system to enhance the targeting effect of tigecycline (TIG) on *Klebsiella pneumoniae*, thereby improving its antibacterial efficacy. **Impact Statement:** This study represents the first report of loading TIG onto hemoglobin (HB) nanoparticles. This system utilizes the iron dependence of *K. pneumoniae* to enhance the targeting ability of TIG and increase the drug concentration at the infection site, thereby enhancing the antibacterial activity and providing a promising strategy for combating drug-resistant gram-negative bacterial pneumonia. **Introduction:** *K. pneumoniae* is a gram-negative bacillus that causes severe primary pneumonia with high pathogenicity and increasing drug resistance. TIG is a key therapeutic option, but its clinical effectiveness is limited by extensive systemic distribution and insufficient drug concentration at infectious foci. HB serves as a promising protein nanocarrier and provides iron, an essential nutrient for *K. pneumoniae* growth, suggesting great potential for targeted antibiotic delivery. **Methods:** Molecular docking was performed to analyze the binding affinity and interaction between HB and TIG. TIG–HBNP was fabricated via a drug–protein self-assembly approach. The nanoparticles were characterized in terms of particle size, zeta potential, drug-loading efficiency, morphology using transmission electron microscopy and atomic force microscopy, stability, and biocompatibility. Targeting capability was evaluated. Pharmacokinetic profiles and antibacterial activity were further assessed. **Results:** Molecular docking verified stable binding between TIG and HB. TIG–HBNP exhibited a uniform spherical morphology, a particle size of approximately 200 nm, a negative surface charge, and a drug-loading efficiency exceeding 20%. The nanoparticles showed favorable stability and safety. Enhanced targeting to *K. pneumoniae* was confirmed in both in vitro and in vivo models. Improved pharmacokinetic behavior and enhanced antibacterial activity against *K. pneumoniae* were also observed. **Conclusion:** TIG–HBNP enhances TIG’s therapeutic efficacy against *K. pneumoniae* infections. Furthermore, HB holds promise as a versatile carrier for diverse antibiotics, offering a scalable platform to combat multidrug-resistant pathogens.

## Introduction

*Klebsiella pneumoniae* is a major opportunistic pathogen and the second most common species in the Enterobacteriaceae family. It is widely found in soil, water, plants, and hospital environments, causing various infections in humans and animals [1,2]. Notably, *K. pneumoniae* is one of the few gram-negative bacilli that can cause primary pneumonia and is a leading cause of hospital-acquired pneumonia [3,4]. Despite antibiotic treatment, the mortality rate remains high—often exceeding 50% in immunocompromised patients. In recent decades, the emergence of hypervirulent and multidrug-resistant (MDR) strains has exacerbated its clinical burden [5,6], highlighting the urgent need for new therapeutic strategies against *K. pneumoniae* infections.

At present, β-lactam and quinolone antibiotics are widely used to treat *K. pneumoniae* infections in livestock and poultry [7]. In human clinical practice, 8 major classes of antibiotics are commonly employed: carbapenems (e.g., meropenem), tetracyclines (e.g., tigecycline [TIG]), polypeptides (e.g., polymyxins), fluoroquinolones, cephamycins, aminoglycosides, penicillins, and cephalosporins [8]. The use of ceftazidime/avibactam, polymyxins, and TIG, either alone or in combination, has become a salvage therapy option for combating MDR and carbapenem-resistant *K. pneumoniae* strains. Among these, TIG possesses broad-spectrum antibacterial activity, effective against nearly all gram-positive and gram-negative bacteria [9,10]. It has become regarded as a “last-resort” antibiotic [11]. In clinical practice, TIG is mainly administered intravenously for the treatment of pneumonia caused by *K. pneumoniae*. Its antibacterial activity is achieved through reversible binding to the 30S ribosomal subunit in bacteria, thereby obstructing the attachment of aminoacyl-tRNA to the A site. This inhibition prevents the incorporation of amino acids into the elongating peptide chain and subsequently suppresses bacterial protein synthesis and proliferation [12]. However, intravenous administration results in the systemic distribution of TIG, with limited penetration into infection sites [13]. On the other hand, TIG demonstrates a low ratio of pulmonary alveolar epithelial fluid to plasma concentration, resulting in poor penetration into the extracellular pulmonary compartment. The drug concentration at 12 h postinfusion often remains subtherapeutic for eradicating gram-negative bacilli. While dose escalation could theoretically enhance local drug concentrations, it is constrained by dose-dependent gastrointestinal toxicity [14]. Thus, targeted delivery strategies that concentrate TIG at infection sites hold promise to maximize therapeutic efficacy against *K. pneumoniae* while minimizing systemic toxicity.

In recent years, with the advancement of nanotechnology, polymer nanoparticles (NPs) have developed into advantageous carriers for antibiotic delivery, capable of achieving effective encapsulation or surface attachment of antibacterial compounds [15–18]. Polymer NPs can provide several advantages for antibiotics, such as facilitating the targeting of antibiotics to pathogens, improving the pharmacokinetics and biodistribution of antibiotics, overcoming cell and tissue barriers, enhancing antibacterial efficacy, and improving resistance [19–22]. Various nanocarriers have demonstrated broad-spectrum antibacterial activity against various gram-positive bacteria, gram-negative bacteria, and other microorganisms [19,23]. Among them, protein-based polymer NPs have attracted much attention due to their biocompatibility and biodegradability [24–28]. For clinical applications, effective antibacterial nanomedicines must maintain stability in serum to prevent rapid clearance by the immune system, facilitate penetration of the vascular barrier, and ensure targeted delivery to the infection site [29,30]. Preliminary investigations revealed that *K. pneumoniae* requires iron for growth [31], and hemoglobin (HB)—the predominant iron-carrying protein in red blood cells—is involved in oxygen transport via its central iron atom [32]. HB can also be internalized into monocytes and macrophages through CD163 receptor-mediated endocytosis, supporting its potential to be an effective targeted delivery carrier. Moreover, the drug release profile of HB can be modulated via structural or environmental alterations, enabling controlled, sustained drug delivery for continuous therapeutic effect. Additionally, HB possesses unique oxygen-carrying capabilities and a well-established safety profile. Notably, the HB–haptoglobin complex can be recognized and cleared by organs including the liver, spleen, and bone marrow, which confers favorable biocompatibility and degradability while minimizing adverse reactions and immunogenicity [33].

Leveraging these insights, we engineered a tigecycline–hemoglobin (TIG–HBNP) system to improve the TIG targeting of *K. pneumoniae*, with the aim of improving its antibacterial efficacy. Initially, molecular docking simulations were employed to assess the binding mode and affinity between HB and TIG, confirming that TIG could stably interact with HB. Subsequently, optimal conditions for hemoglobin nanoparticle (HBNP) synthesis were established, and TIG–HBNP was fabricated utilizing a “drug–protein self-assembly” strategy involving HB and TIG. The synthesized TIG–HBNP underwent comprehensive characterization via laser particle size analysis, transmission electron microscopy, and atomic force microscopy. Drug release kinetics were evaluated using a dialysis method. The targeting capability of TIG–HBNP for *K. pneumoniae* was validated in an in vivo bacterial model employing mice infected with *K. pneumoniae*, with results demonstrating effective targeting. In addition, the in vivo biodistribution, pharmacokinetics, and antibacterial efficacy of TIG–HBNP were investigated, confirming robust antibacterial activity against *K. pneumoniae*. Collectively, these data validate the HB-based biomimetic nanoplatform as a potent strategy to augment therapeutic efficacy of TIG against *K. pneumoniae* infections. Furthermore, HB holds potential as a multifunctional carrier for the delivery of other antibiotics, providing a broad platform to address MDR bacterial pathogens.

## Results

### Conformational screening of TIG and proteins and molecular dynamics characterization of the TIG–HB complex

Molecular docking is an important tool for predicting the binding mode and affinity between small-molecule compounds and target proteins. It provides a scientific basis for screening potential active compounds. In this study, molecular docking was performed using Vina to evaluate the potential binding between TIG and HB.

Further, molecular dynamics (MD) simulations were conducted for TIG and HB. The phenolic hydroxyl group and secondary amine on the TIG benzene ring formed 4 hydrogen bonds with the amino acid residues C/Lys99 (~3.5 Å), D/Glu100 (~2.5 Å, ~2.8 Å), and D/Asn107 (~3.0 Å) in the HB subunits C and D. TIG’s tetracene and *tert*-butyl could form hydrophobic interactions with the amino acid residues A/Pro95 (~4.0 Å) and D/Lys103 (~3.8 Å) of the HB subunits A and D. These results indicate that TIG can form strong intermolecular forces with HB.

**Fig. 1.**
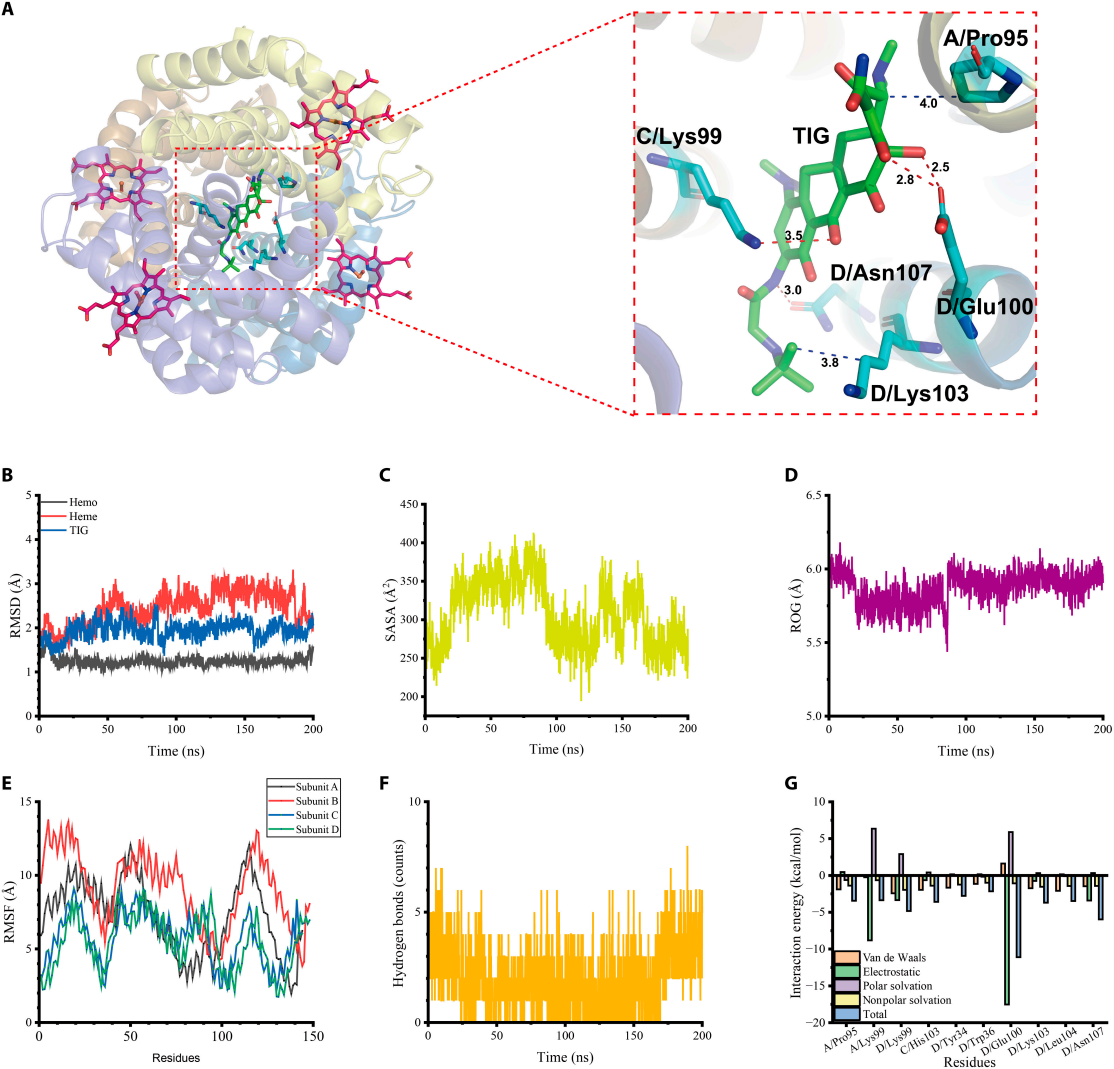
Docking and kinetic analysis of tigecycline (TIG) with hemoglobin (HB) molecules. (A) Molecular docking of TIG with HB. (B) Root mean square deviation (RMSD) values of protein backbone atoms. (C) Solvent-accessible surface area (SASA) reduction indicating increased structural compactness. (D) Radius of gyration (ROG) showing converged folding. (E) Per-residue root mean square fluctuations (RMSFs). (F) Intermolecular hydrogen bond occupancy. (G) Molecular mechanics/Poisson–Boltzmann surface area (MM/PBSA)-derived binding energy decomposition (units: kcal/mol).

To comprehensively assess the stability of the TIG–HB complex, MD simulations were performed to evaluate system stability, solvent accessibility, protein conformational compactness, fluctuations of residues relative to their average positions, and interactions of the TIG–HB complex by using indicators such as root mean square deviation, solvent-accessible surface area, radius of gyration, root mean square fluctuations of compounds, and hydrogen bond analysis. The root mean square deviation values of HB, heme, and TIG fluctuated at ~1.2, ~2.5, and ~1.8 Å (Fig. 1B), while the solvent-accessible surface area and radius of gyration exhibited progressive reductions (Fig. 1C and D), indicating enhanced structural compactness. The root mean square fluctuations were small, and the peaks were all below 15 Å (Fig. 1E). These indicate that TIG and HB are stable throughout the simulation process, with an increase in compactness, and are able to form a stable combination. Additionally, hydrogen bond analysis showed that the number of hydrogen bonds in TIG–HB was 0 to 6, indicating the presence of hydrogen bond interactions (Fig. 1F).

In order to better explain the interaction energy between HB and TIG, we used molecular mechanics/generalized Born surface area to determine the binding energy of the TIG–HB complex at the equilibrium stage. As shown in Fig. 1G, binding energy decomposition revealed that TIG could bind to the key residues of HB. Summarized with binding energy, the dominant contributions from van der Waals forces (−24.6393 kcal/mol) and electrostatic interactions (−32.3296 kcal/mol), were partially offset by the polar solvation energy (+12.7226 kcal/mol). The nonpolar solvation energy was −19.0158 kcal/mol, and the total binding energy was −62.3144 kcal/mol. The results indicate stable TIG–HB complex formation, and the main interaction is van der Waals interaction.

### Preparation and characterization of TIG–HBNP

**Fig. 2.**
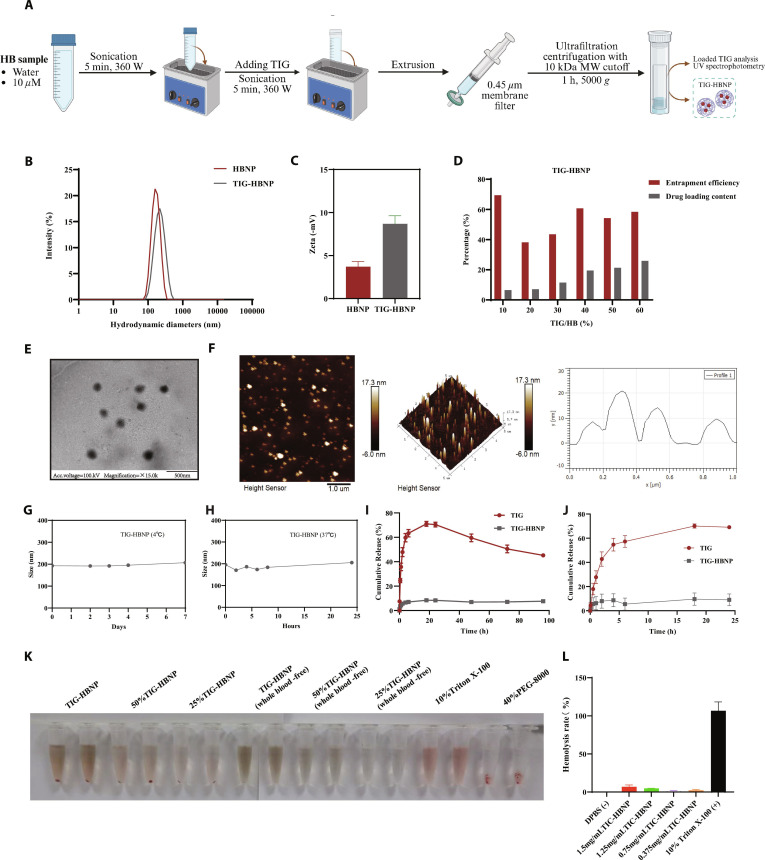
Preparation and characterization of tigecycline–hemoglobin nanoparticle (TIG–HBNP). (A) Schematic diagram of the preparation process. (B) Particle size and polymer dispersity index (PDI) graph. (C) Zeta potential. (D) Encapsulation rate and drug loading. (E) Transmission electron microscope image. (F) Atomic force microscope image. (G) 7-d stability observation. (H) 24-h stability observation. (I) 96-h in vitro release curve (*n* = 3). (J) 24-h in vitro release curve (*n* = 3). (K) Hemolysis test. (L) Hemolysis rate of TIG–HBNP. MW, molecular weight; UV, ultraviolet.

The natural activity of HB (especially the structure containing Fe^2+^/Fe^3+^) is the molecular basis for the interaction between HB and *K. pneumoniae*. Due to this issue, we developed a simple and easy method for the formation of HBNPs that minimize protein denaturation, and a synthetic illustration is shown in Fig. 2A. The HBNPs were facilely constructed by dissolving human HB in Milli-Q water and stirred under ultrasonic conditions. Under optimized conditions (HB concentration: 10 μM; HB/TIG feed ratio: 40%, w/w; Fig. S1A and B), TIG was successfully encapsulated into HBNPs using an identical protocol. As shown in Fig. 2B, the average hydrodynamic diameters of HBNP/TIG–HBNP determined by dynamic light scattering were 183.45 ± 3.62 and 192.82 ± 7.13 nm, respectively, indicating suitability as nanocarriers. TIG–HBNP exhibited more negative charges (−8.69 ± 0.94 mV) on the particle surface compared with HBNPs (−3.78 ± 0.70 mV), indicating the binding between TIG and HBNPs and that the negative charges contributed to the stabilities via repulsive forces (Fig. 2C). When the drug feeding ratio was 40% (TIG/HB), the drug-loading content (%) was 22.48 for TIG–HBNP, and the average hydrodynamic diameter of TIG–HBNP was below 200 nm. Correspondingly, the encapsulation efficiency (%) was calculated at 58.92 for TIG–HBNP (Fig. 2D). Transmission electron microscopy further confirmed that TIG–HBNP was spherical (Fig. 2E). The surface morphology and roughness of HBNPs in the empty and laden states were estimated from the atomic force microscope images via contact mode. In comparison with the empty HBNPs, TIG–HBNP showed a larger diameter with a more uniform and smoother surface. This likely arises from TIG-mediated self-assembly of HB through electrostatic and hydrogen bonding interactions of functional groups related to both TIG and the surface of HBNPs (Fig. 2F and Fig. S2). The particle size of TIG–HBNP stored at 4 and 37 °C for different durations demonstrated the long-term stability of HBNP nanocarriers (Fig. 2G and H). The drug release behaviors of TIG–HBNP were evaluated by the dialysis method. The in vitro drug release profiles demonstrated that TIG–HBNP achieved only ~10% burst release within 24 h, with cumulative release plateauing at <15% over 96 h (Fig. 2I and J), confirming negligible premature leakage.

Next, we evaluated the safety of TIG–HBNP using a hemolysis test. As shown in Fig. 2K, the 10% Triton X-100 group had a distinct red color, indicating complete hemolysis. Meanwhile, the 40% PEG-8000 negative control group had no obvious red color in the supernatant, and there was red blood cell precipitation at the bottom, indicating that no hemolysis occurred. Overall observation indicated that the samples in the TIG–HBNP group were slightly blood red, but the hemolysis degree of all samples containing TIG–HBNP was much lower than that of the positive control group. Although the supernatants of the TIG–HBNP, 25% TIG–HBNP, and 50% TIG–HBNP groups showed a trend of changing from transparent to yellowish-red turbidity as the concentration of TIG–HBNP increased, red blood cell precipitation could be clearly observed after centrifugation in these groups. Compared with the corresponding groups of each concentration of TIG–HBNP (whole-blood-free [WB-free]), the color of the supernatants was similar. This suggests that the color of the supernatants of the TIG–HBNP, 25% TIG–HBNP, and 50% TIG–HBNP groups may not be from hemolysis of red blood cells but mainly caused by the color of HB. Moreover, by comparing the hemolysis rate results (Fig. 2L), it was found that when the concentration of TIG–HBNP was 1.5 mg/ml, the average hemolysis rate was 6.92%. Further examination of the average hemolysis rate at 1.25 mg/ml resulted in a rate of 4.79%. Therefore, when the concentration of TIG–HBNP was less than 1.25 mg/ml, the hemolysis rate was always <5%. This indicates that TIG–HBNP has good biocompatibility.

### The in vitro antibacterial effect of HBNP and its targeting of *K. pneumoniae*

**Fig. 3.**
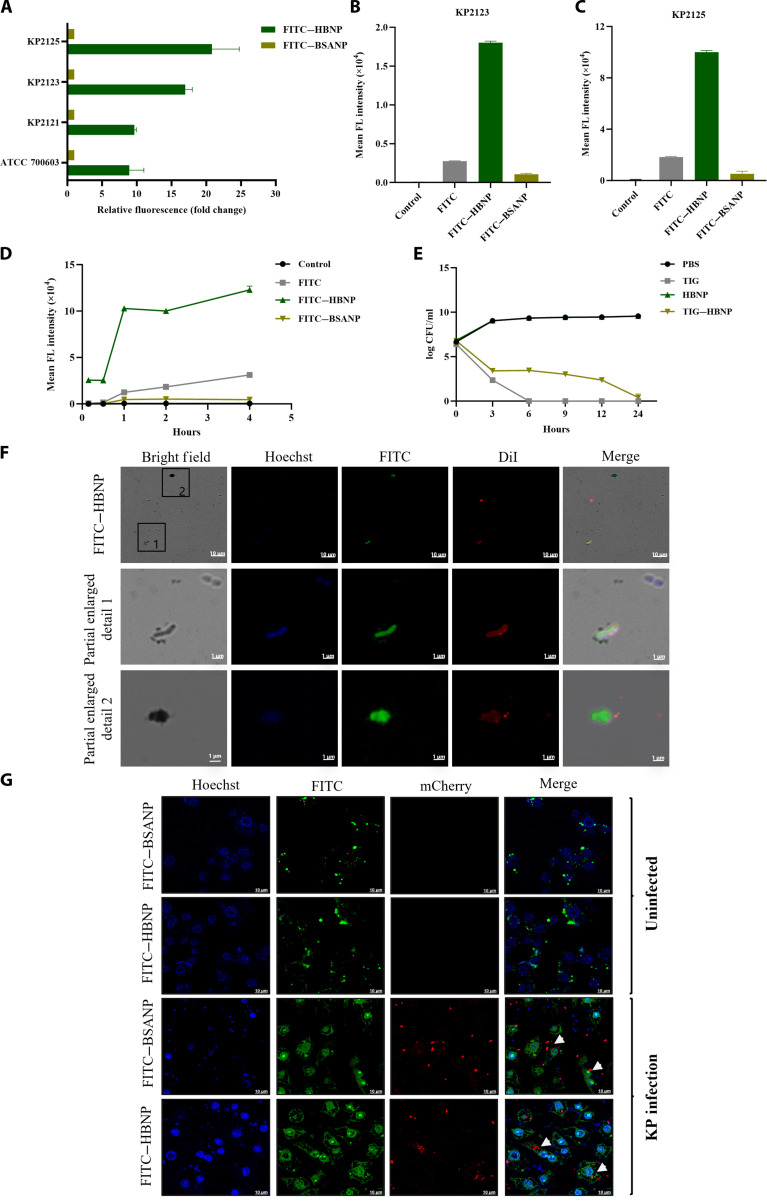
In vitro interaction between *Klebsiella pneumoniae* and nanoparticles. (A) The fluorescence intensity of bacterial strains (KP2125, KP2123, KP2121, and ATCC 700603) incubating with fluorescein isothiocyanate (FITC)–HBNP or FITC–bovine serum albumin nanoparticle (BSANP) for 2 h. (B) The fluorescence intensity of KP2123 incubating with FITC–HBNP or FITC–BSANP. (C) The fluorescence intensity of KP2125 incubating with FITC–HBNP or FITC–BSANP. (D) The fluorescence intensity of KP2125 incubating with FITC–HBNP or FITC–BSANP for different durations. (E) The in vitro antibacterial time curve of TIG–HBNP. (F) The targeting ability of FITC–HBNP to *K. pneumoniae*. (G) After incubating mouse macrophage RAW 264.7 (uninfected/infected with KP2125 overnight) with FITC–HBNP and FITC–BSANP for 15 min, the fluorescence distribution position was observed through confocal microscopy. The white arrow indicates the location of the bacteria within the cells. FL, fluorescence; PBS, phosphate-buffered saline; Dil, 1,1′-dioctadecyl-3,3,3′,3′-tetramethylindocarbocyanine perchlorate.

To assess the interactions between the HBNP and *K. pneumoniae* in vitro, 4 *K. pneumoniae* subtypes (KP2121, KP2123, KP2125, and ATCC 700603) were used to co-incubate with HBNPs or bovine serum albumin nanoparticles (BSANPs) that conjugated with fluorescein isothiocyanate (FITC) and detected their interactions using flow cytometry. From the results, it can be seen that HBNPs interact with all 4 subtypes of *K. pneumoniae*, with the strains of the KP2123 and KP2125 subtypes exhibiting the most significant interaction with HBNP. In contrast, BSANPs did not show any signs of interaction with the 4 strains (Fig. 3A to C). Further experiments showed that the interaction between HBNP and *K. pneumoniae* was most significant after 1 h of co-incubation and persisted for up to 4 h (Fig. 3D). In vitro antibacterial experiments showed that the minimum inhibitory concentration (MIC) of TIG–HBNP is 3.25 μg/ml, and both TIG–HBNP and free TIG completely inhibited the growth of *K. pneumoniae* after 24 h. However, it is worth noting that the rate at which HBNP inhibits *K. pneumoniae* is significantly lower than that of TIG (Fig. 3E).

For evaluating the in vitro targeting efficacy of HBNPs on cells infected by *K. pneumoniae*, we used a laser confocal microscope to evaluate the specific targeting ability of FITC–HBNP to *K. pneumoniae*. FITC–HBNP and FITC–BSANP were incubated with KP2125, respectively. It was observed that a high-intensity green fluorescence signal appeared inside the bacteria incubated with FITC–HBNP (Fig. 3F), and there was some FITC–HBNP adhered to the outside of the bacteria (indicated by white arrows in Fig. 3G). A certain green fluorescence signal was observed inside the bacteria incubated with FITC–HBNP, while no obvious fluorescence signal was observed inside the bacteria incubated with FITC–BSANP (Fig. S3A). These results show that HB specifically aggregated around or within the bacteria, and the formation of HBNPs enhanced this interaction. This indicates that HBNP has a specific targeting effect on *K. pneumoniae*. HB after being prepared into NPs not only retains the characteristics that allow it to be recognized by the bacteria but also exhibits a more powerful binding and endocytosis effect.

Furthermore, in order to investigate whether HBNPs can effectively enter macrophages and bind to *K. pneumoniae*, we established an in vitro cell infection model and used laser scanning confocal microscopy to observe the localization of HBNP and *K. pneumoniae* within the cells. The results are shown in Fig. 3G. The FITC-labeled HBNP and mCherry-labeled *K. pneumoniae* were found to be colocalized within the cells (indicated by the white arrow). There was no obvious colocalization between FITC-labeled BSANPs and *K. pneumoniae* (Fig. S3B). These results indicate that HBNP and *K. pneumoniae* also interact with each other within the cells. These results indicate that HBNPs can not only bind to extracellular *K. pneumoniae* but also be phagocytosed by immune cells and enter the cells to interact with *K. pneumoniae*, preliminarily demonstrating the targeting effect of HBNPs on *K. pneumoniae*.

### The ability of HBNPs to target *K. pneumoniae* and antibacterial activity in vivo

**Fig. 4.**
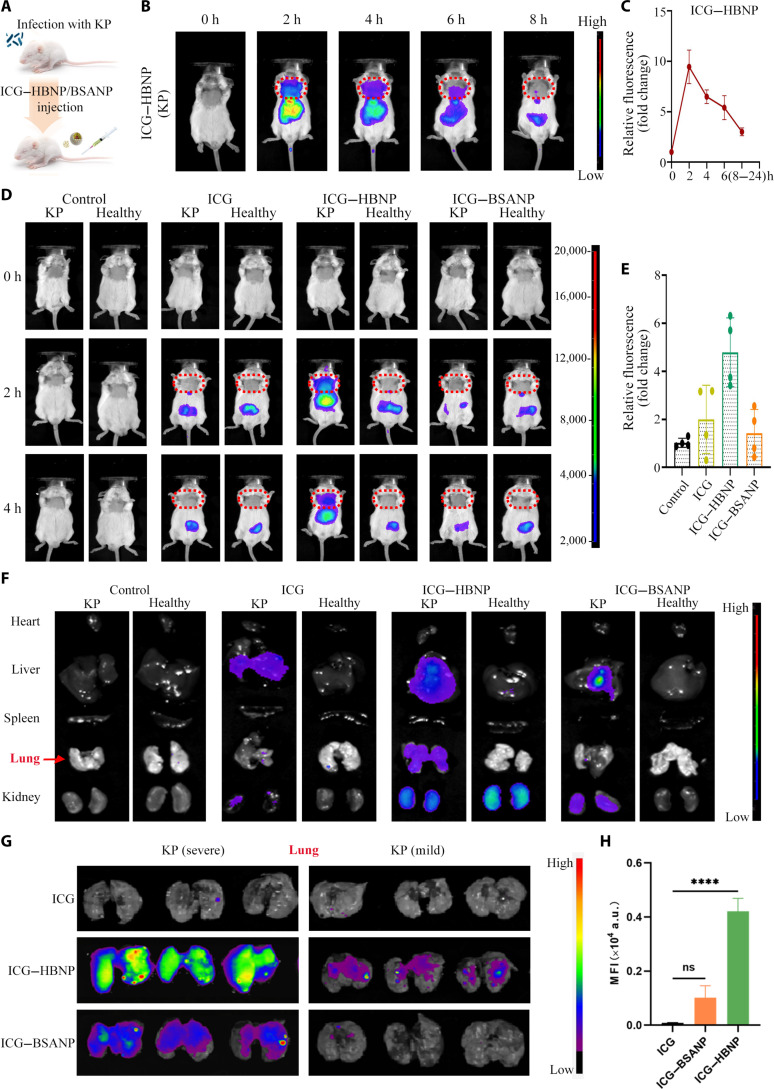
In vivo distribution and targeting effect on *K. pneumoniae* of HBNPs/BSANPs. (A) Schematic diagram of the experimental procedure. (B and C) Live fluorescence images and fluorescence intensities of *K. pneumoniae* mice 0 to 8 h after injection of indocyanine green (ICG)–HBNP nanoparticles. (D and E) Live fluorescence images and fluorescence intensities of *K. pneumoniae* mice and normal mice 0 to 4 h after injection of PBS, ICG, ICG–HBNP, and ICG–BSANP nanoparticles. (F) Fluorescence images of major organs of *K. pneumoniae* mice and normal mice 4 h after injection of PBS, ICG, ICG–HBNP, and ICG–BSANP nanoparticles. (G and H) Fluorescence images and fluorescence intensities of the lungs of *K. pneumoniae* mice and normal mice 4 h after injection of ICG, ICG–HBNP, and ICG–BSANP nanoparticles. MFI, mean fluorescence intensity; ns, not significant.

We further explored whether HBNPs can target *K. pneumoniae* in vivo. After infecting mice with *K. pneumoniae*, HBNPs or BSANPs that conjugated with indocyanine green (ICG) were administered via intravenous injection, and the distribution of ICG–HBNP and ICG–BSANP in the mice was measured by a live fluorescence imager (Fig. 4A). ICG–HBNP was observed to exhibit maximal fluorescence accumulation in the lungs of mice at 2 h, after which the signal gradually weakened until it declined to near-background levels by 8 h (Fig. 4B and C). Interestingly, no localized signal enrichment of ICG–HBNP was detected in the lungs of healthy mice. The infection-dependent pulmonary accumulation confirms active bacterial targeting rather than passive organ retention. No fluorescence signal aggregation of ICG–BSANP was observed in either healthy mice or mice infected with *K. pneumoniae*, further indicating that HBNPs, rather than BSANPs, actively target *K. pneumoniae* in vivo (Fig. 4D and E and Fig. S4). After dissection of the mice, the fluorescence signals were detected, revealing that both ICG–HBNP and ICG–BSANP were initially accumulated in the liver (Fig. 4F), which was consistent with many nanomedicine-based drug delivery systems. In addition, stronger fluorescent signals were detected in the lungs of severely infected mice than in the lungs of mildly infected mice (Fig. 4G and H). This indicates that the number of HBNP increases in areas with higher densities of *K. pneumoniae*. These results further confirm the bacteria-targeting ability of HBNPs, which actively target *K. pneumoniae*.

### In vivo pharmacokinetic and pharmacological studies of TIG–HBNP

**Fig. 5.**
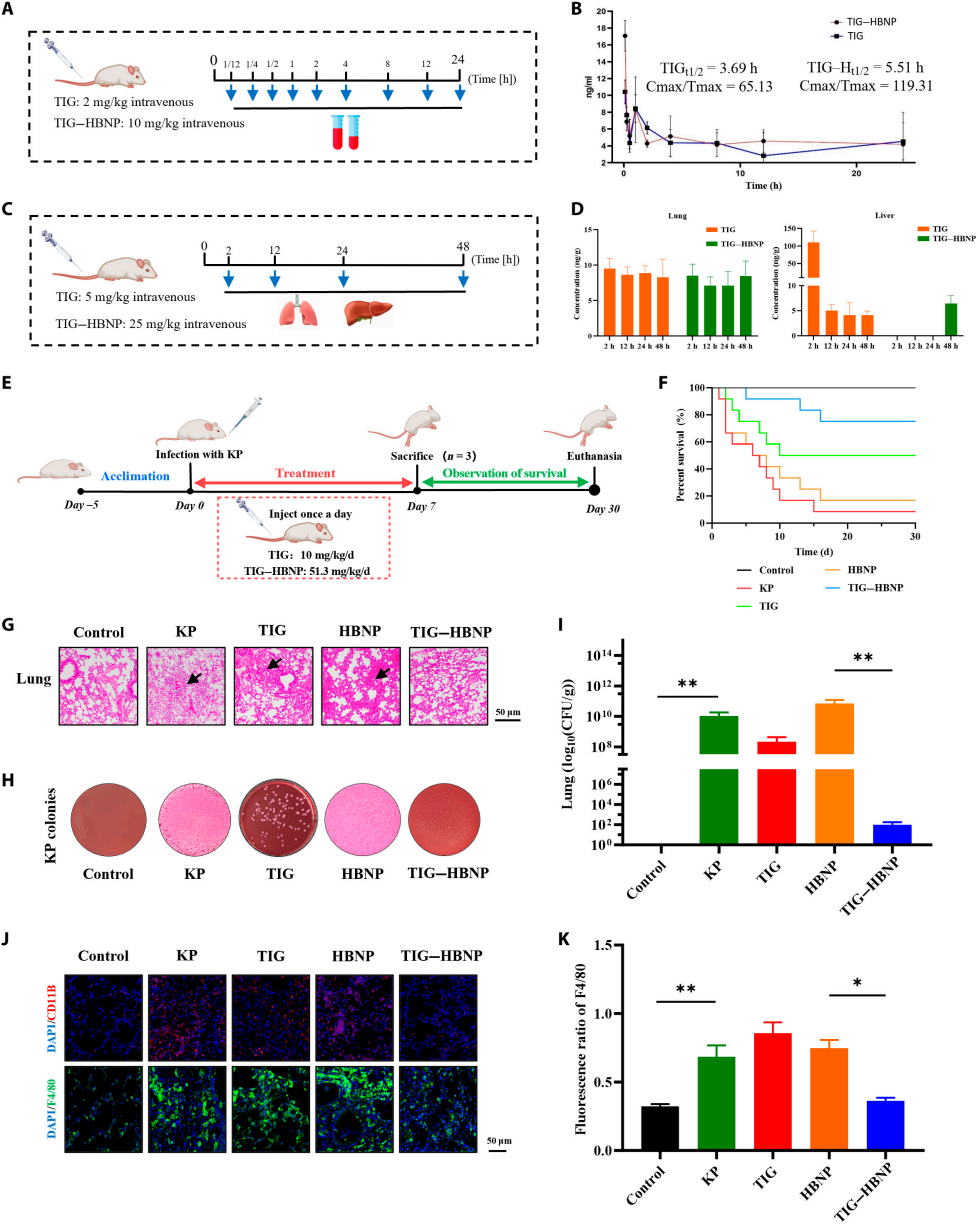
In vivo pharmacological efficacy research of TIG–HBNP. (A) Schematic diagram of the pharmacokinetic experiment process. (B) Pharmacokinetic detection of TIG–HBNP (*n* = 6). (C) Schematic diagram of the drug metabolism experiment process. (D) Metabolic distribution of TIG–HBNP in mouse liver and lung tissues (*n* = 6). (E) Pharmacodynamic experiment process schematic diagram. (F) Changes in mouse mortality rate (this experiment independently used 12 mice). (G) Histopathology of livers in mice (*n* = 3, scale = 50 μm). (H) Qualitative detection of *K. pneumoniae* in mouse lung tissue (*n* = 3). (I) Quantitative analysis of *K. pneumoniae* in mouse lung tissue (*n* = 3, log_10_ CFU of *K. pneumoniae* per gram of lung). (J) Immunofluorescence detection of mouse lung tissue (*n* = 3, scale = 50 μm). (K) Immunofluorescence intensity of mouse lung tissue (*n* = 3). DAPI, 4′,6-diamidino-2-phenylindole.

Quantitative determination of TIG in blood samples from the mouse tail vein at different time points was performed using liquid chromatography–tandem mass spectrometry (LC–MS/MS) (Fig. 5A). The pharmacokinetic curves showed that the half-life (t1/2) of TIG–HBNP extended to 5.51 h, compared to 3.69 h for TIG alone, demonstrating that TIG–HBNP had a prolonged drug release. On the other hand, comparison of release rates showed a slightly faster release rate for TIG–HBNP compared to those for TIG (Fig. 5B). Further detection of TIG distribution in mice injected (intravenously) with TIG and TIG–HBNP (Fig. 5C) revealed that high concentrations of TIG were found in the lung tissue of both the TIG and TIG–HBNP groups, which persisted for up to 48 h. The concentration of TIG in liver tissue gradually decreased after 2 h, suggesting that the metabolic process of TIG–HBNP in liver was basically completed at 48 h (Fig. 5D).

To further confirm the antibacterial effect of TIG–HBNP, mice infected with *K. pneumoniae* were treated with TIG and TIG–HBNP injections (intravenous) (Fig. 5E). Compared with those of the TIG group and HBNP group, the survival rate of TIG–HBNP group mice was significantly increased (Fig. 5F). The results of pathological examination also confirmed that TIG–HBNP improved the edema and injury of lung tissue, and the effect was better than that of the TIG alone (Fig. 5G and Fig. S5). Consistently, detection of bacterial colonies in the mouse lung showed that TIG–HBNP significantly inhibited the growth of *K. pneumoniae* in the lung compared to the TIG and HBNP groups (Fig. 5H and I and Fig. S6). In addition, we performed indirect immunofluorescence detection of mouse lung tissue using CD11B antibody (Fig. 5J and Fig. S7). The results showed that TIG–HBNP could reduce the infiltration of immune cells in the lung tissue. In particular, indirect immunofluorescence detection of mouse lung tissue using F4/80 antibody showed that the number of macrophages in the lung tissue of mice was significantly increased (*P* < 0.01) due to *K. pneumoniae* infection, while TIG–HBNP treatment could reduce the number of macrophages (*P* < 0.05) (Fig. 5K).

### The biological safety of TIG–HBNP

**Fig. 6.**
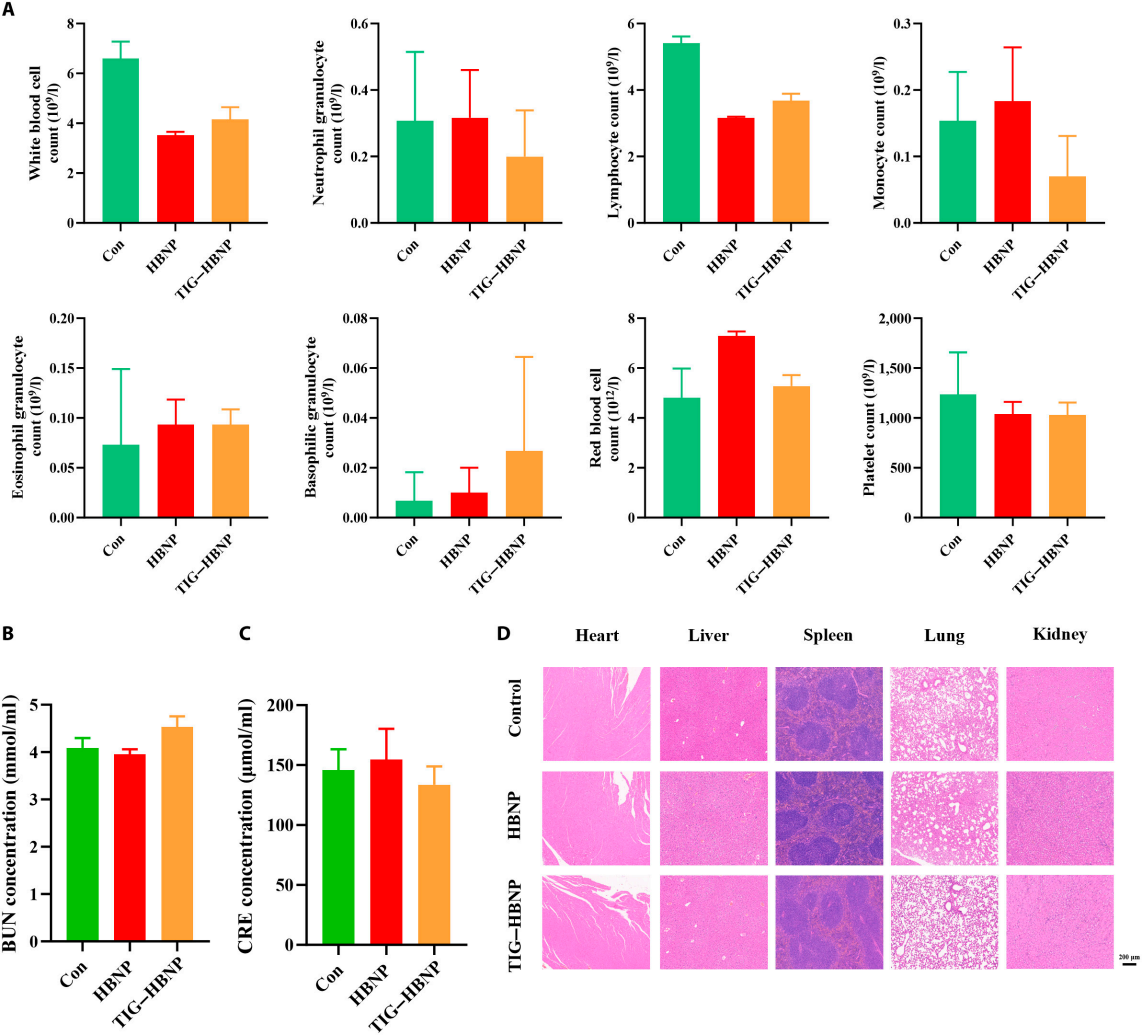
TIG–HBNP safety evaluation. (A) Blood index values in mice treated with saline solution, HBNPs, and TIG–HBNP (*n* = 3). (B) Blood urea nitrogen (BUN) concentration in mice treated with saline solution, HBNPs, and TIG–HBNP (*n* = 3). (C) Creatinine (CRE) concentration in mice treated with saline solution, HBNPs, and TIG–HBNP (*n* = 3). (D) Hematoxylin–eosin (HE) staining of the heart, liver, spleen, lung, and kidney. Scale bar: 200 μm (*n* = 3).

The biological safety of a formulation is important for its application. We conducted an assessment of the biological safety of TIG–HBNP. We administered HBNP and TIG–HBNP to healthy mice via tail vein injection. Blood samples were collated for blood index values analysis and kidney function markers such as urea nitrogen (blood urea nitrogen [BUN]) and creatinine (CRE). The 5 internal organs were subjected to hematoxylin–eosin (HE) staining. As shown in Fig. 6, after mice had been administered HBNPs and TIG–HBNP, there were no significant differences between their blood index values, BUN, and CRE and those of the control group mice (Fig. 6A and B). Furthermore, all were within the normal range. This confirmed their good safety. Additionally, the HE staining results of the heart, liver, spleen, lung, and kidney further demonstrated their safety (Fig. 6D and Fig. S8).

## Discussion

With the increasing prevalence of antimicrobial resistance in *K. pneumoniae*, TIG is often administered as a “last-resort” antibiotic [34,35]. The focus of this study was to design an HBNP nano-delivery system loaded with TIG for targeted treatment of *K. pneumoniae*, thereby enhancing therapeutic efficacy through targeted drug delivery. Structurally, HB is a tetramer composed of 2 α and 2 β peptide chains forming a central cavity, thereby providing a rigid scaffold and sufficient space for small-molecule encapsulation [36–39]. Nanomaterial-related HB-based oxygen carriers have been studied in hemorrhagic shock, ischemic stroke, cancer, and wound healing [40–44]. In this study, molecular docking analyses indicated that among the several commonly used protein carriers, only HB and TIG had binding poses. Further MD simulations revealed that the amino groups of HB can engage in intermolecular interactions with various structural motifs on TIG. Additionally, the fabrication procedure for the nanoplatform is relatively straightforward and can be performed under ambient conditions, leading to the generation of a nanoscale carrier with high drug-loading capacity and safety. The prepared TIG–HBNP demonstrated prolonged drug release behavior and favorable biocompatibility, thus establishing a solid basis for further translational and clinical application.

The prolonged half-life of TIG facilitates its widespread distribution throughout various tissues postinjection; however, this nonspecific distribution limits its efficient accumulation at the lesion site, often causing elevated drug concentrations in healthy tissues and promoting drug resistance, which may lead to suboptimal clinical outcomes [34,35]. Iron is a vital element for bacterial proliferation and biofilm formation [45], with the central heme group of HB tightly binding Fe^2+^ [46]. Hence, HB was selected as a natural targeting carrier in this study. Results from our experiments demonstrate that the association between HB and *K. pneumoniae* is highly specific rather than random, nonspecific protein–bacteria adsorption. This specific HB–*K. pneumoniae* interaction is evident both intracellularly and extracellularly. Furthermore, HB exhibits a stronger affinity for drug-resistant and highly virulent strains of *K. pneumoniae*, highlighting its efficacy in delivering therapeutics specifically to infection sites. The entry of HBNPs into the bacterial cytoplasm enables efficient antibacterial activity. Additionally, in vivo fluorescence imaging demonstrated that HBNPs target *K. pneumoniae* specifically rather than healthy mouse lung tissue, and the accumulation of HBNPs increased proportionally with *K. pneumoniae* density, further confirming effective targeting capability.

In mice infected with *K. pneumoniae*, treatment with TIG–HBNP led to a significant reduction in bacterial lung burden, reflecting strong therapeutic efficacy. At the same time, the lung tissue damage caused by *K. pneumoniae* infection was recovered, indicating the therapeutic success. Notably, TIG–HBNP extended the release profile of TIG and decelerated its metabolic clearance in mice. As previously discussed [47–50], the intrinsic clearance mechanism of free HB supports the design of HB-modified nanomaterials to target M2-type macrophages endogenously, signifying unique potential in treating macrophage-related inflammatory diseases. Indirect immunofluorescence assays of lung tissues from *K. pneumoniae*-infected mice revealed that compared to free TIG, TIG–HBNP not only diminished bacterial counts but also decreased macrophage infiltration, resulting in reduced lung tissue damage. Furthermore, after administering the drug to healthy mice for 7 d, the blood index values, BUN, CRE, and HE staining of the 5 internal organs were analyzed. Combined with the weight changes in the mice from the pharmacological experiment and the in vitro hemolysis rate, it can be concluded that the TIG–HBNP has high biological safety.

Although this strategy demonstrated efficacy in treating *K. pneumoniae*-induced pneumonia, further steps are necessary prior to clinical translation of these polymeric NPs. As part of routine internal quality control, TIG–HBNP underwent a 7-d preliminary evaluation; however, comprehensive stability assessments, including accelerated and long-term studies, remain essential. Furthermore, while evidence supports the specific targeting of *K. pneumoniae* by HBNPs, the precise mechanistic details underpinning this phenomenon require clarification. Postinjection, TIG–HBNP predominantly accumulates in the lungs, but its distribution across discrete lung regions and within various cell types warrants further investigation.

## Materials and Methods

### Materials

TIG was purchased from Tianjin Siansi Biochemical Technology Co., LTD. HB (F2411218), ICG, FITC, and formic acid (mass spectrometer grade) were purchased from Shanghai Aladdin Biochemical Technology Co., Ltd. (Shanghai, China). Bovine serum albumin (BSA) and phosphate-buffered saline (PBS; D6501) was purchased from Shanghai Macklin Biochemical Co., Ltd. (Shanghai, China). MacConkey agar medium was purchased from Qingdao Rishui Bio-Technology Co., Ltd. (Qingdao, China). Methanol and acetonitrile (high-performance liquid chromatography [HPLC]–MS grade) were purchased from Thermo Fischer Scientific (Shanghai, China). Fetal bovine serum (10099141C) was purchased from Invitrogen Inc. (USA). PBS buffer (1×, pH 7.2 to 7.4) (C8020), and Hoechst 33342 staining solution (C8053) were purchased from Shanghai Titan Technology Co., Ltd. (Shanghai, China). A bicinchoninic acid protein assay kit, 4% paraformaldehyde, anti-CD11b rabbit polyclonal antibody (anti-CD11b antibody, GB115689), anti-F4/80 rat monoclonal antibody (anti-F4/80 antibody, GB12027), Alexa Fluor 594-labeled goat anti-rabbit immunoglobulin G (IgG) (GB28301), Alexa Fluor 488-labeled goat anti-rabbit IgG (GB25303), 4′,6-diamidino-2-phenylindole (DAPI) staining reagent (G1012), and antifluorescence quenching sealant (G1401) were purchased from Wuhan Servicebio Technology Co., Ltd. (Wuhan, China). The cell membrane red fluorescent staining kit (1,1′-dioctadecyl-3,3,3′,3′-tetramethylindocarbocyanine perchlorate [DiI]) and 10% Triton X-100 solution (ST797) were purchased from Shanghai Biyuntian Biotechnology Co., Ltd. (Shanghai, China). Cyanide methemoglobin standard solution (27042) was purchased from Zhongshan Research Institute of Tianjin Modern High Tech Research Institute (Tianjin, China). PEG-8000 solution (40%; SL9514) was purchased from Beijing Coolaber Science & Technology Co., Ltd. (Beijing, China). The HB test solution test kit (C021-1-1), the urea nitrogen (BUN) reagent kit (C013-2-1), and the CRE reagent kit (C011-2-1) were purchased from NanJing JianCheng Bioengineering Institute (Nanjing, China).

### Bacteria, cell lines, and animals

*K. pneumoniae* was provided by Henan Agricultural University: (a) *K. pneumoniae* ATCC 700603 (standard strain/control group), (b) KP2121 (experimental strain 3/hypervirulent, resistance 2.0 group), (c) KP2123 (experimental strain 2/resistant 4.0 group), and (d) KP2125 (experimental strain 1/hypervirulent experimental group).

Murine macrophages cell line RAW 264.7 (ATCC TIB-71) was purchased from Wuhan Punosay Life Technology Co., LTD. (Wuhan, China). Cells were cultured in Dulbecco’s modified Eagle medium containing 10% fetal bovine serum at 37 °C with 5% CO_2_ in a humidified incubator.

Specific-pathogen-free (SPF) Kunming (KM) mice (20 ± 2 g, 6 to 8 weeks old) were purchased from Hunan Sleek Jingda Experimental Animal Co., Ltd. (Hunan, China), and the quality was qualified (AQCN-430727240103804871). The animals were maintained under SPF conditions in a single room with a 12-h light/dark cycle. The feeding facilities are licensed by the Chongqing Municipal Laboratory Animal Management Committee (SYXK(Chongqing)2020-0006). Animal protocols complied with ethical regulations for animal experimentation and were approved by the Institutional Animal Care and Use Committee of Southwest University (IACUC-20200215-01).

### Molecular docking analysis of TIG interactions with diverse HB proteins

To investigate the potential binding interactions between TIG and HB proteins with a possible drug-loading capacity, molecular docking simulations were performed [51]. The 3-dimensional structures of the receptor proteins were obtained from the Protein Data Bank (ID: 2QSP; resolution: 1.85 Å). Water molecules and ions were removed, missing residues were repaired, hydrogens were added, and charges were assigned. The molecular structures of the ligand TIG were retrieved from PubChem, followed by energy minimization, charge assignment, and identification of rotatable bonds. The binding site located in the hollow of the HB protein consisted of 4 subunits, which was defined by PyMOL with the GetBox plugin [52,53]. Docking simulation was carried out with AutoDock Vina, and the optimal docked conformation was used for subsequent molecular dynamics simulation. Similarly, we selected another HB protein (ID: 5E29; resolution: 1.85 Å) and redocked the cocrystal ligand by Vina; the redocked conformation aligned with the cocrystal ligand well, which meant that the docking method could apply to this study.

### MD simulation for the HB catalysis of TIG

We used the AMBER ff99SB-ILDN force field and the general AMBER force field to generate parameters and topologies for HB and TIG, respectively. The distance (greater than 10 Å) between each atom of HB and the simulation box was set. The TIP3P water molecule model was adopted, and Na^+^ and Cl^−^ counterions were used to replace the water molecules to make the simulation system electrically neutral. The entire system was optimized by using the steepest descent method, and 100 ps of NVT ensemble and NPT ensemble was conducted under conditions of 300 K and 1 bar, respectively. Subsequently, 200 ns of MD simulation was performed under periodic boundary conditions, using the V-rescale and Parrinello–Rahman methods to control the temperature (300 K) and pressure (1 bar). The Newtonian equations of motion were calculated using the transition integral method, with a time step of 2 fs. Long-range electrostatic interactions were calculated using the particle mesh Ewald method, with a Fourier spacing of 1.6 Å. All bond lengths were constrained using the LINear Constraint Solver algorithm. The binding free energy decomposition of the compound was calculated using molecular mechanics/Poisson–Boltzmann surface area.

### Preparation of TIG–HB

Based on the protocol established by Zhang et al. [54], we optimized the process and screened the NP preparation method as follows: the HB concentration (10 to 80 μM) was screened through particle size; 6 distinct molar ratios of HB:TIG (10% to 60%, w/w) were set up, and TIG–HBNP were prepared. The preparation process is shown in Fig. 2A.

Free-drug concentrations were determined via using an ultraviolet spectrophotometer to measure the concentration of TIG in the lower layer of the filtrate after ultrafiltration. The drug concentration in the drug-loaded HB solution was obtained by conversion, thereby calculating the drug encapsulation rate and drug-loading amount. The calculation formulas for the encapsulation rate and drug-loading amount are as follows:

$$\text{Encapsulation rate}=\left(W_{\text{drug}}-W_{\text{free}}\right)/W_{\text{drug}}\times 100\%,$$ (1)

$$\begin{array}{c} \text{Drug}-\text{loading rate}=\left(W_{\text{drug}}-W_{\text{free}}\right)/\\ \left[\left(W_{\text{drug}}-W_{\text{free}}\right)+W_{\text{carrier}}\right]\times 100\%. \end{array}$$ (2)

*W*_drug_ is the total amount of drug input, *W*_free_ is the unencapsulated drug amount, and *W*_carrier_ is the total amount of the carriers invested.

### Characterization of TIG–HBNP

The particle size and polymer dispersity index were determined using a BeNano 90 nanosize analyzer (Dandong Baotai Instrument Co., Ltd., China). Zeta potential measurements were acquired via using a ZEV3600-Zeta potential analyzer (Malvern, UK).

The morphology of the samples was observed using a JEM-2100F high-resolution transmission electron microscope (Lorentz, Japan), operating at a 100-kV accelerating voltage. The newly prepared drug-loaded NPs were dropped onto a copper sheet and then stained with 2% (w/v) phosphotungstic acid solution for 60 s, after carefully removing excess solution, and the samples were naturally dried at room temperature before being tested.

The conformation and 3-dimensional morphology of the TIG–HBNP were observed using a Dimension FastScan/Icon atomic force microscope (Bruker, Germany); 2.0 μl of the drug-loaded NP solution (concentration of 50.0 μg/ml) was dropped onto a freshly dissociated mica sheet and left to air-dry at room temperature. The observation was conducted in intermittent contact mode at a temperature of 25 °C.

### In vitro stability and release profiles

1. NP aliquots were incubated in constant-temperature environments of 4 and 37 °C. The particle size changes of the NPs were measured continuously for 7 d at 4 °C and for 24 h at 37 °C to evaluate their stability.
2. The drug release behavior of TIG–HBNP was evaluated by dialysis. The specific procedure is as follows:
  a. One milliliter of TIG–HBNP (1 mg/ml) was added to a dialysis bag (molecular weight: 3.5 kDa), and dialysis was performed in 19 ml of PBS (pH 7.4) under continuous horizontal shaking (60 rpm) at 37 °C.
  b. At specified time points (5 min, 30 min, 1 h, 2 h, 4 h, 6 h, 18 h, and 24 h), 1 ml of the release medium was taken and the same volume of the release medium was immediately added.
  c. A TBD2000 ultraviolet spectrophotometer was used to measure the concentration of TIG in the release medium and the cumulative release percentage of TIG from the TIG–HBNP was calculated. Experiments were conducted in quintuplicate (*n* = 5) with independent biological replicates.

### In vitro hemolysis test

We investigated the in vitro hemolysis of different concentrations of TIG–HBNP to assess its safety. Rabbit WB (2 ml) was diluted to a total HB concentration of 10 ± 1 mg/ml, and then the diluted WB was mixed with TIG–HBNP (1.5 mg/ml, calculated based on the drug loading), 50% TIG–HBNP, 25% TIG–HBNP, TIG–HBNP (without WB), 50% TIG–HBNP (without WB), and 25% TIG–HBNP (without WB). Triton X-100 solution (10%) was used as the positive control for hemolysis, and 40% PEG-8000 solution was used as the negative control without hemolysis. After incubation at 37 °C in a constant-temperature and constant-humidity incubator for 3 h, the mixture was centrifuged at 800 × g for 15 min, and the absorbance of the supernatant was measured at 540 nm. Finally, the hemolysis rate was calculated using the formula HR = PFH sample/TBHd × 100% (HR, hemolysis percentage; PFH sample, sample peripheral free hemoglobin concentration, in units of milligrams per milliliter; TBHd, HB concentration diluted to (10 ± 1) mg/ml, in units of milligrams per milliliter).

### Flow cytometry detection

The in vitro uptake of HBNPs by *K. pneumoniae* was detected using a FACSVerse fluorescence flow cytometer (Becton Dickinson, USA). HB and BSA were labeled with FITC. Free FITC, FITC–HBNP, and FITC–BSANP were co-cultured with the ATCC 700603 strain, KP2121 strain, KP2123 strain, and KP2125 strain for 2 h. The reaction was terminated by placing the sample on ice. The bacteria were collected by centrifugation (6,000 × g, 8 min). Unbound FITC–protein was removed by washing 3 times with 1× PBS. The bacteria were resuspended with an appropriate amount (1 ml) of PBS, and the fluorescence signal of the bacteria was detected by flow cytometry (fluorescence channel: FITC; excitation wavelength: 488 nm; emission wavelength: 530 nm).

### Specific targeting capability analysis of HBNPs and *K. pneumoniae*

The binding specificity of HBNP to *K. pneumoniae* was evaluated using a STELLARIS 5 laser scanning confocal microscope (Leica, Germany). The operation method was as follows: The density of *K. pneumoniae* (KP2125) was adjusted to OD_600_ of 0.2 in PBS, and then FITC–HBNP was added to a concentration of 100 μg/ml. After incubation at 37 °C for 1 h, the bacterial suspension was washed 3 times with PBS buffer. Bacteria were subsequently stained with PBS containing Hoechst 33342 (1×) and DiI dye (1×) at 37 °C for 30 min. After another 3 washes with PBS, bacterial cells were fixed with 4% paraformaldehyde for 10 min. Samples were mounted onto confocal dishes using an antifade mounting medium and visualized under a laser scanning confocal microscope.

### In vitro cell culture and bacterial infection experiments

For evaluating the in vitro targeting efficacy of HBNPs on cells infected by *K. pneumoniae*, RAW 264.7 cells were seeded at a density of 1 × 10^5^ cells per well in a laser confocal culture dish and cultured at 37 °C and 5% CO_2_ for 12 h. Upon achieving ~90% confluency of the cells, 100 μl of *K. pneumoniae* (KP2125) (1 × 10^5^ CFU/ml) was added for co-culture for 24 h. Meanwhile, cells without bacterial infection were set as the control. The cells were washed with 1× PBS buffer 3 times and then supplemented with complete Dulbecco’s modified Eagle medium containing 10 μg/ml FITC-labeled HB (HBNPs) or BSA. The cells were incubated in the dark for 15 min. Subsequently, the cells were washed with 1× PBS buffer 3 times, the FITC–HBNP and FITC–BSA that did not bind were removed, and 2 ml of the complete cell culture medium was added. Then, the laser confocal culture dish was placed under an ultrahigh-resolution laser confocal microscope to observe the distribution of FITC–HBNP and FITC–BSA in the cells, as well as their colocalization with *K. pneumoniae*.

### The MIC of TIG–HBNP

The MIC of TIG–HBNP was determined using the CLSI M07-A11 standard broth microdilution method. After the target strain was revived and passaged, it was inoculated into cation-adjusted Mueller–Hinton broth (CAMHB) and cultured at 37 °C until the logarithmic growth phase (OD_600_ ≈ 0.4 to 0.6). Then, the CAMHB was diluted to a working bacterial suspension of 5 × 10^5^ CFU/ml. The TIG–HBNP stock solution was serially diluted in a 96-well plate, with 50 μl of the drug solution and 50 μl of the bacterial suspension added to each well, resulting in a final volume of 100 μl. At the same time, free TIG was set as the positive control. After 16 to 18 h of static incubation at 37 °C, the lowest drug concentration that completely inhibited bacterial growth was determined based on the absence of visible turbidity. All experiments were performed with 3 replicates.

### In vivo fluorescence imaging of TIG–HBNP

For the establishment of the *K. pneumoniae* infection mouse model (Fig. 4A), all mice were anesthetized via chloroform inhalation. After anesthesia, mice were maintained in a vertical position with the head upright and inoculated intranasally with 40 μl of *K. pneumoniae* suspension (at a concentration of 3 × 10^7^ CFU/ml). Mice in the control group received an equal volume of PBS buffer in the same way. After inoculation, mice were held in the head-up position for 20 s and subsequently rotated to distribute the inoculum. After waking up, the mice were placed in cages and allowed to eat freely. Successful model establishment was confirmed by the presence of the following symptoms within 1 to 2 d: increased respiratory rate, reduced locomotor activity, ruffled fur, and increased periocular secretions.

ICG was used to label HB and BSA, respectively. Pneumonia model mice received intravenous injections of ICG–HBNP, ICG–BSANP, or free ICG via the tail vein. Fluorescence accumulation in the heart, liver, spleen, lungs, and kidneys at predetermined time points was monitored using an in vivo imaging system. Mice were autopsied when necessary, and organs (heart, liver, spleen, lungs, and kidneys) were harvested and subjected to fluorescence quantification using a whole-body optical bioimaging system (FOBI).

### In vivo pharmacokinetic experiments

The operation process of in vivo pharmacokinetic animal experiments is shown in Fig. 5A. Seventy-eight SPF KM mice were acclimatized for 5 d and randomly divided into the TIG and TIG–HBNP groups (*n* = 39 per group). Both formulations (TIG solution vs. TIG–HBNP suspension) were administered intravenously into the tail vein of the mice in each group. The injection concentration was 0.2 mg/ml (normalized to equivalent TIG dosages), and the dosage was 2 mg/kg. Blood samples of 0.7 ml were collected from the orbital area of 3 mice in each group at 5 min, 15 min, 30 min, 1 h, 2 h, 4 h, 8 h, 12 h, 24 h, 48 h, 72 h, and 96 h after administration. The blood samples were collected into polypropylene microcentrifuge tubes pre-chilled with K_2_EDTA (pre-cooled in ice water bath). Samples were immediately centrifuged at 3,000 × g for 10 min (4 °C) to obtain plasma, which was then quickly placed in a −80 °C refrigerator for subsequent drug metabolism detection.

### Tissue distribution study

The operation process of in vivo pharmacokinetic animal experiments is shown in Fig. 5C. Forty-eight SPF KM mice (equal sex distribution) were divided into the TIG group and the TIG–HBNP group (*n* = 6 per group, 3 males and 3 females). After 5 d of adaptive feeding, the test substances TIG and TIG–HBNP were injected into the tail vein in each group. The injection concentration was 0.625 mg/ml (calculated based on the TIG content), and the dosage was 5 mg/kg. Blood samples were collected from each group at 2, 12, 24, and 48 h after administration. After the last blood collection, liver and lung tissues were taken, rapidly frozen in liquid nitrogen, and stored at −80 °C until bioanalysis.

### Detection of TIG under LC–MS conditions

The collected plasma, liver, and lung tissues were homogenized with methanol:acetonitrile (1:1) solution, respectively. After centrifugation at 12,000 × g for 10 min (4 °C), the supernatant containing TIG was collected. Aliquots (3 μl) of the supernatant were injected into the LC–MS system. When analyte concentrations exceeded the linear range of the calibration curve, samples were diluted with a blank matrix prior to protein precipitation.

The liquid-phase system was a Shimadzu high-performance liquid chromatograph (Shimadzu HPLC, Kyoto, Japan) equipped with an Ultimate AQ-C18 column (4.6 mm × 150 mm, 5 μm; Welch Technology Corporation, Boston, MA, USA) at 30 °C. Methanol (A) and 0.1% formic acid (B) were used as the mobile phase with the following gradient program: 0 to 5 min, 1% to 1% (A); 5 to 10 min, 1% to 3% (A); and 10 to 20 min, 3% to 8% (A). The flow rate of the mobile phase was 1 ml/min, and the injection volume was 3 μl.

Quantitative measurements were carried out using the AB SCIEX API 4000 triple quadrupole system. Detection employed electrospray ionization in negative ion mode with multiple reaction monitoring. The optimal conditions were as follows: capillary voltage 5,500 V, gas flow rate 5 l/min, dry gas temperature 600 °C, sheath gas temperature 600 °C, dry gas flow rate 3 l/min, and nebulizer pressure 55 psi; nitrogen gas was used during the instrument operation. The TIG ions had an *m*/*z* of 586.3/513.2, a voltage of 80 V, and collision energies of 29 eV. Data were collected using the Analyst TF (version 1.6.1) software and analyzed by the MultiQuant (version 1.5) software.

### In vivo protective effect of TIG–HBNP

A study design schematic illustrating the in vivo protective efficacy assessment is shown in Fig. 5E. Thirty SPF KM mice were randomly divided into 5 groups (*n* = 6 in each group): the control group, model group, TIG treatment group, HBNP treatment group, and TIG–HBNP treatment group. Following an established protocol (see the “In vivo fluorescence imaging of TIG–HBNP” section) for 1 h, the mice were administered the drugs through tail vein injection. The control group and model group were given 200 μl of PBS buffer solution. The TIG group was given 10 mg/kg TIG solution (200 μl in volume). The TIG–HBNP group was given 51.3 mg/kg TIG–HBNP solution (containing 10 mg/kg of TIG). The HBNP group was given the same amount of HBNP solution. The treatment was carried out for 7 d, and the survival status and weight changes of the mice were observed and recorded daily. Lung tissues were taken for observing gross pathological changes, performing HE staining, and determining bacterial load by plating serial dilutions of lung homogenates.

Deceased mice or those euthanized at the 7-d endpoint underwent dissection, and the right lung tissues were taken for the colony count experiment. The colony count experiment was performed as follows: sterile thoracotomy was performed, and the lung tissue was removed and rinsed with sterile normal saline. The right lung tissue was blotted dry of surface water and weighed and homogenized in 1 ml of PBS; 100 μl of each was applied to MacConkey agar plates and evenly spread with glass beads. Plates were incubated at 37 °C for 24 h before bacterial counts were performed.

HE staining was used to detect cell damage in lung tissue. The specific steps were as follows: part of the lung tissue was fixed with 4% paraformaldehyde. After dehydration with gradient alcohol, clearing with xylene, and paraffin embedding, wax blocks were made and serially cut into 4-μm-thick sections. The sections were baked at 60 °C for 30 min and then deparaffinized with xylene. Xylene was removed with different concentrations of ethanol (100%, 95%, 90%, and 85%). Cell nuclei were stained with hematoxylin for about 2 min, rinsed with running water, and blued with PBS. The cytoplasm was stained with eosin and rinsed with tap water after observation. The slices were dehydrated with the above ascending concentration gradient of ethanol, cleared with xylene, and finally sealed with neutral gum. Stained sections were observed and photographed under a light microscope.

### The survival period of mice after drug treatment

Sixty SPF KM mice were randomized into 5 experimental groups (*n* = 12 in each group): the control group, model group, TIG treatment group, HBNP treatment group, and TIG–HBNP treatment group. The control group and the model group were administered 200 μl of sterile PBS, and the TIG group was administered 10 mg/kg TIG solution (about 200 μl). The TIG–HBNP group was treated with 51.3 mg/kg TIG–HBNP solution (containing TIG 10 mg/kg), and the HBNP group was treated with the same amount of HBNP solution for 7 d. Following completion of the 7-d treatment regimen, animals were maintained under standard conditions for an additional 30 d. The survival of mice was observed daily, and the changes in the survival time of mice after different drug treatments were analyzed. Following an established protocol (see the “In vivo protective effect of TIG–HBNP” section), lung tissues of mice were taken for observing gross pathological changes by HE staining and determining bacterial load by plating serial dilutions of lung homogenates.

### Immunofluorescence staining

The lung tissue was fixed with 4% paraformaldehyde. After dehydration with gradient alcohol, clearing with xylene, and paraffin embedding, wax blocks were made and serially cut into 4-μm-thick sections. The sections were baked at 60 °C for 30 min and then deparaffinized with xylene. Xylene was removed with different concentrations of ethanol (100%, 95%, 90%, and 85%). Antigen repair was performed after hydration. They were blocked with 5% BSA and incubated with primary antibodies (anti-CD11b rabbit polyclonal antibody or anti-F4/80 rat monoclonal antibody, 1:500) overnight at 4 °C. After washing, fluorescent secondary antibodies (Alexa Fluor 594-labeled goat anti-rabbit IgG or Alexa Fluor 488-labeled goat anti-rabbit IgG, 1:1,000) were added and incubated for 1 h at room temperature. After washing with 1× PBS buffer, DAPI was added for nuclear staining. Gradient dehydration was performed using different concentrations of ethanol, and the slides were sealed with antifluorescence quenching sealant after clearing with xylene. Subsequently, the tissue section images were observed and recorded under a BX43 upright epifluorescence microscope (Olympus, Japan). Three to 5 nonoverlapping high-power fields per section were systematically captured. The fluorescence intensity was quantified using the ImageJ software (version 1.54f, USA).

### Safety evaluation of TIG–HBNP in healthy mice

Nine SPF-grade male KM mice were randomly divided into 3 groups: the control group, HBNP treatment group, and TIG–HBNP treatment group. The mice were administered the drug via the tail vein for 7 d. The control group was given 200 μl of PBS buffer. The TIG–HBNP group was given 51.3 mg/kg of TIG–HBNP solution (containing 10 mg/kg of TIG). The HBNP group was given the same amount of HBNP solution. On the seventh day after administration, blood was collected from the mouse orbital cavity. Half of the blood sample was placed in a blood collection tube containing heparin sodium for the detection of blood routine indicators. The other half was placed in a 1.5-ml Eppendorf tube. It was left at 4 °C for 2 h and centrifuged, and the supernatant serum was taken for the detection of BUN and CRE. The organs (heart, liver, spleen, lung, and kidney) were collected for HE staining analysis.

### Statistical analysis

All data are presented as mean ± standard deviation, and all statistical analyses were performed using GraphPad Prism 10. The differences between 2 groups were compared by a *t* test. For more than 2 groups, one-way analysis of variance was used for statistical analysis of multiple comparisons. *P* < 0.05 indicates that the data are significantly different. * denotes *P* < 0.05, ** denotes *P* < 0.01, *** denotes *P* < 0.005, and **** denotes *P* < 0.001.

## Data Availability

Data will be made available on request.

## Supplementary Materials

Supplementary 1 Graphical Abstract Figs. S1 to S8
