# Supplementary material for: A Hemoglobin-Based Nanoparticle Delivery System Enhances the Pharmacokinetics and Efficacy of Tigecycline in Klebsiella pneumoniae Infections
Source: BME Front. 2026 Mar 30;7:0241. doi: 10.34133/bmef.0241 (PMC13033835; doi:10.34133/bmef.0241)
Supplement: Supplementary 1 — Graphical Abstract Figs. S1 to S8 [file bmef.0241.f1.zip › Supplementary Material.docx]

**Supplementary Material**


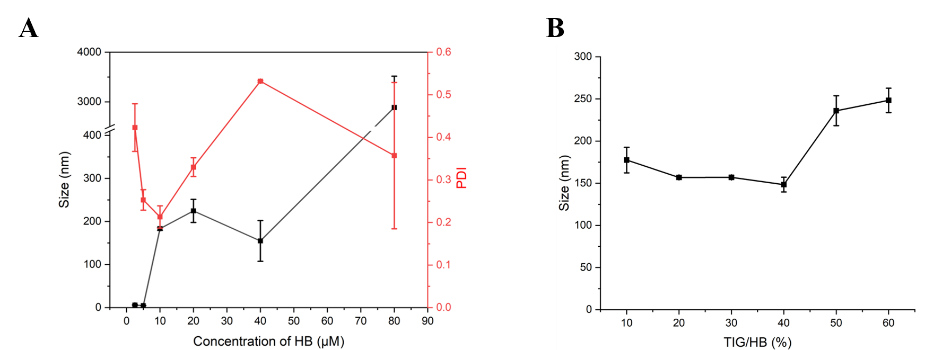


Figure S1. Prescription Optimization Chart

(A) HB concentration screening. (B) Feed ratio (TIG/HB) screening.


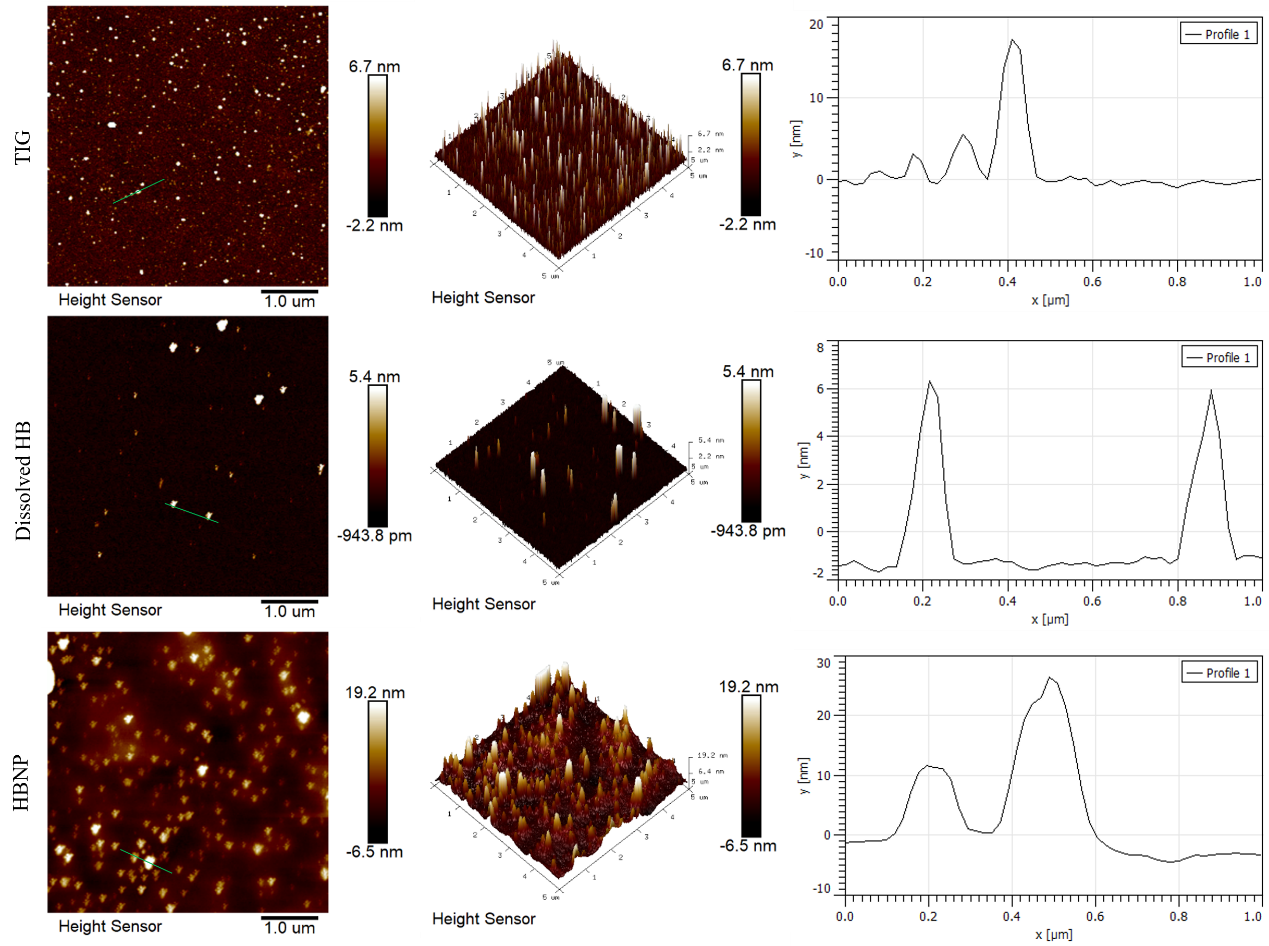


Figure S2. Atomic force microscopy images of TIG, dispersed HB and HBNP without TIG loading —— That is: Supplementary to Fig. 2F.


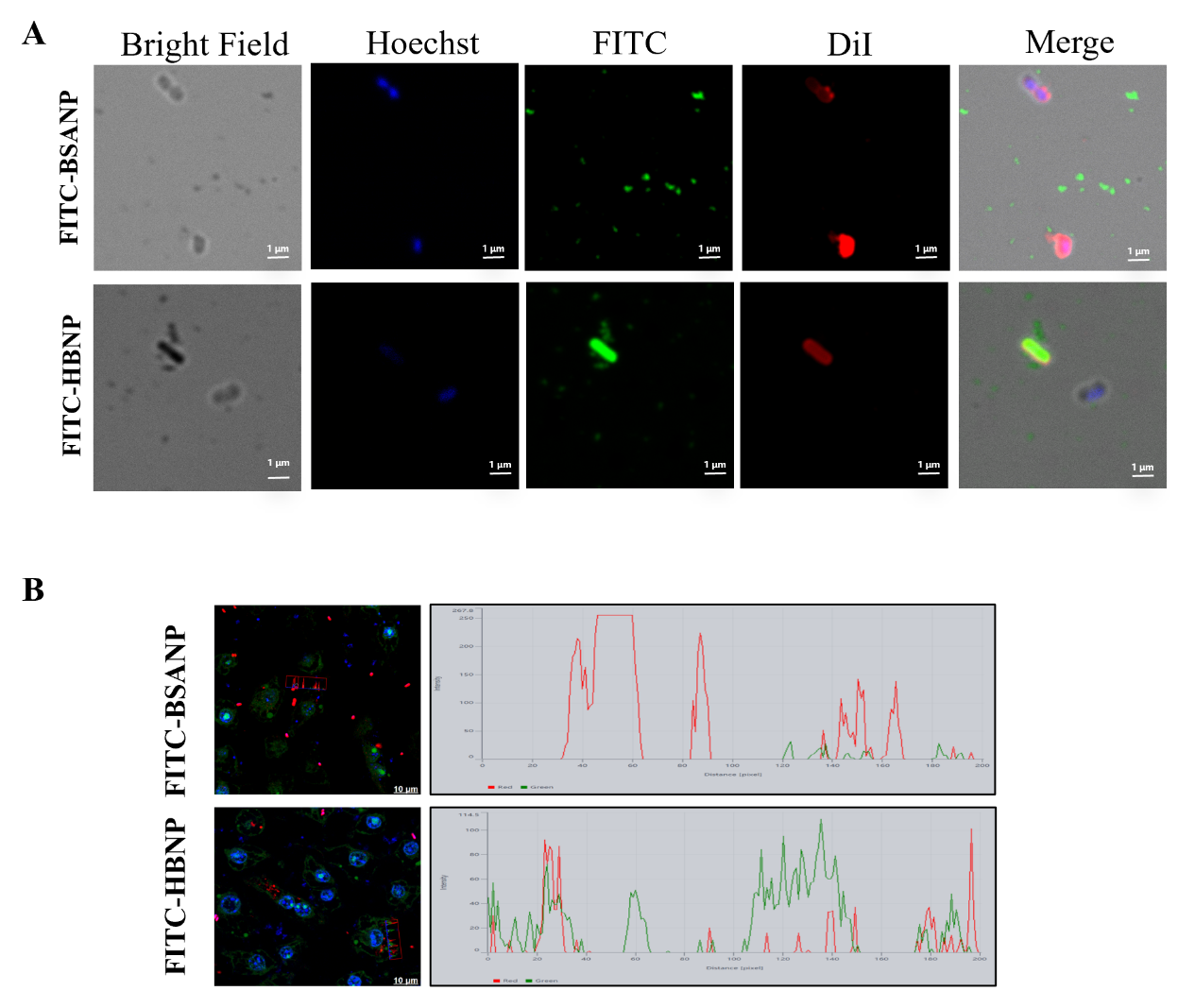


Figure S3. In vitro Comparison of the targeting ability of BASANP and HBNP to *K. pneumoniae*.

(A) After incubating FITC-BSANP and FITC-HBNP with KP2125 respectively, observe the targeting effect of FITC-BSANP and FITC-HBNP on *K. pneumoniae* (scale = 10 μm). (B) Mouse macrophage Raw264.7 was infected with KP2125 overnight, and then FITC-HBNP and FITC-BSANP were co-incubated for 15 minutes. The fluorescence distribution positions were observed through confocal microscopy —— That is: Supplementary to Fig. 3G.


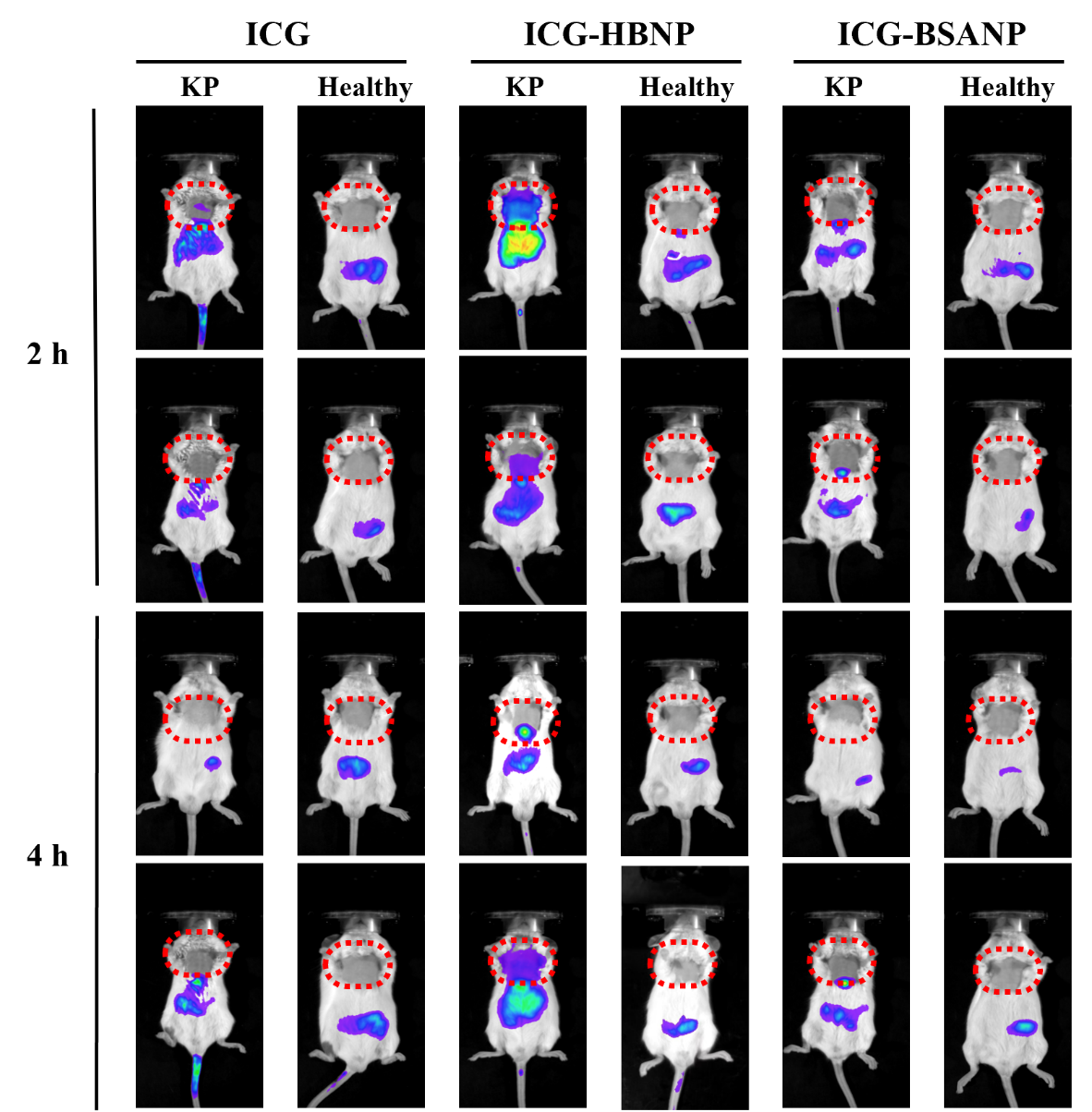


Figure S4. Live fluorescence images of *K. pneumonia* mice and normal mice 0-4 h after injection of ICG, ICG-HBNP and ICG-BSANP —— That is: Supplementary to Fig. 3.


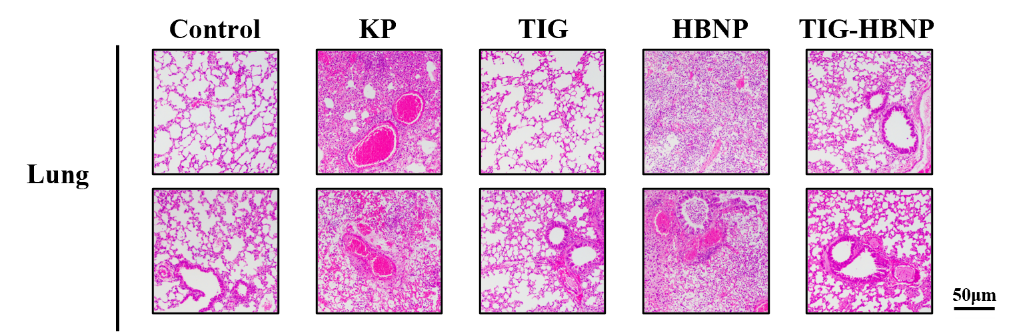


Figure S5. The HE staining results of the lungs of mice infected with *K. pneumoniae* after drug treatment —— That is: Supplementary to Fig. 5G.


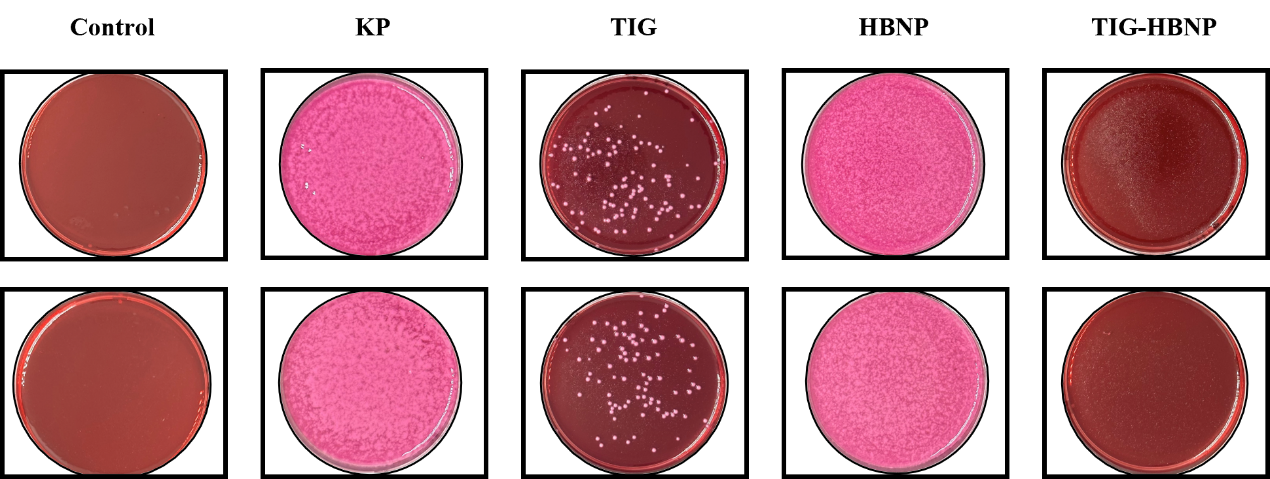


Figure S6. The lungs of mice infected with *K. pneumoniae* and treated with different methods formed representative colonies on the MacConkey agar plate —— That is: Supplementary to Fig. 5H.


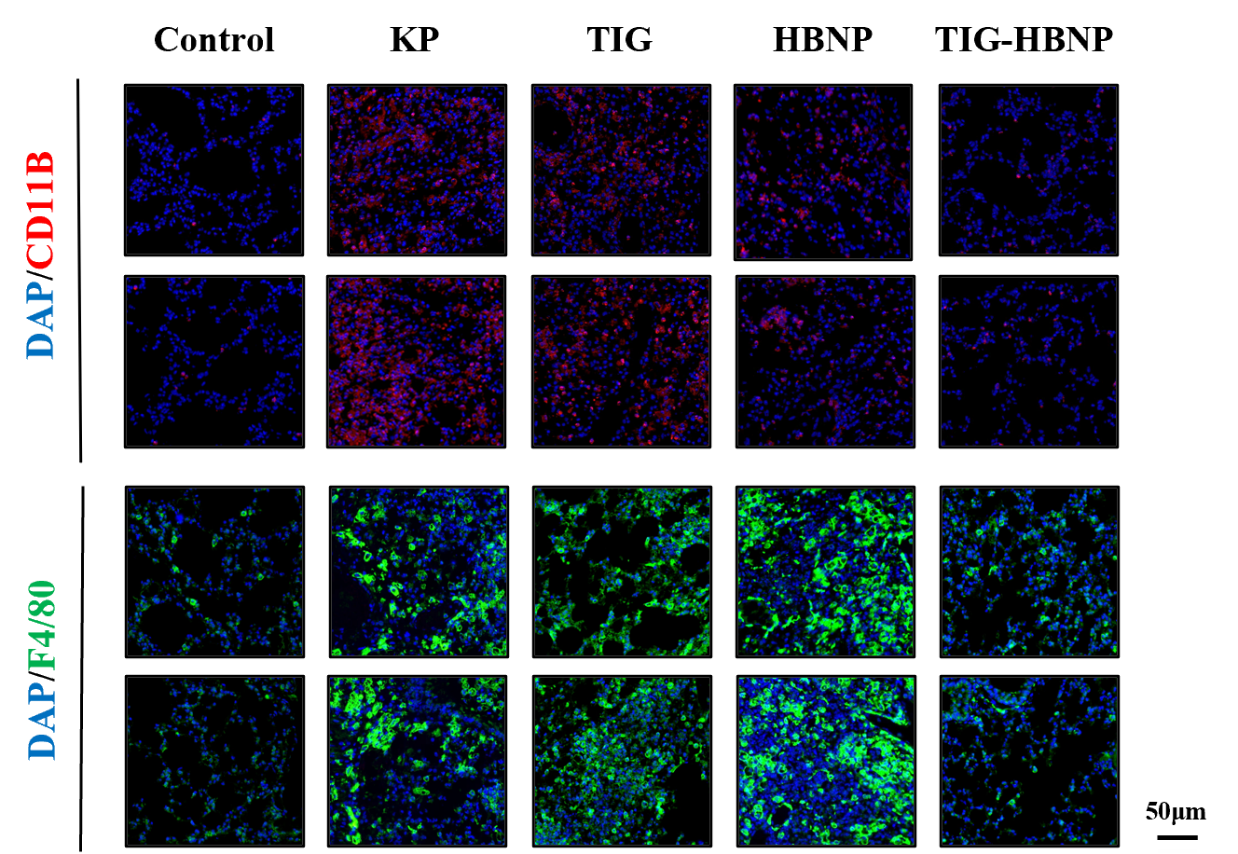


Fig S7. The indirect immunofluorescence detection results of CD11B antibody and F4/80 antibody on mouse lung tissue —— That is: Supplementary to Fig. 5J.


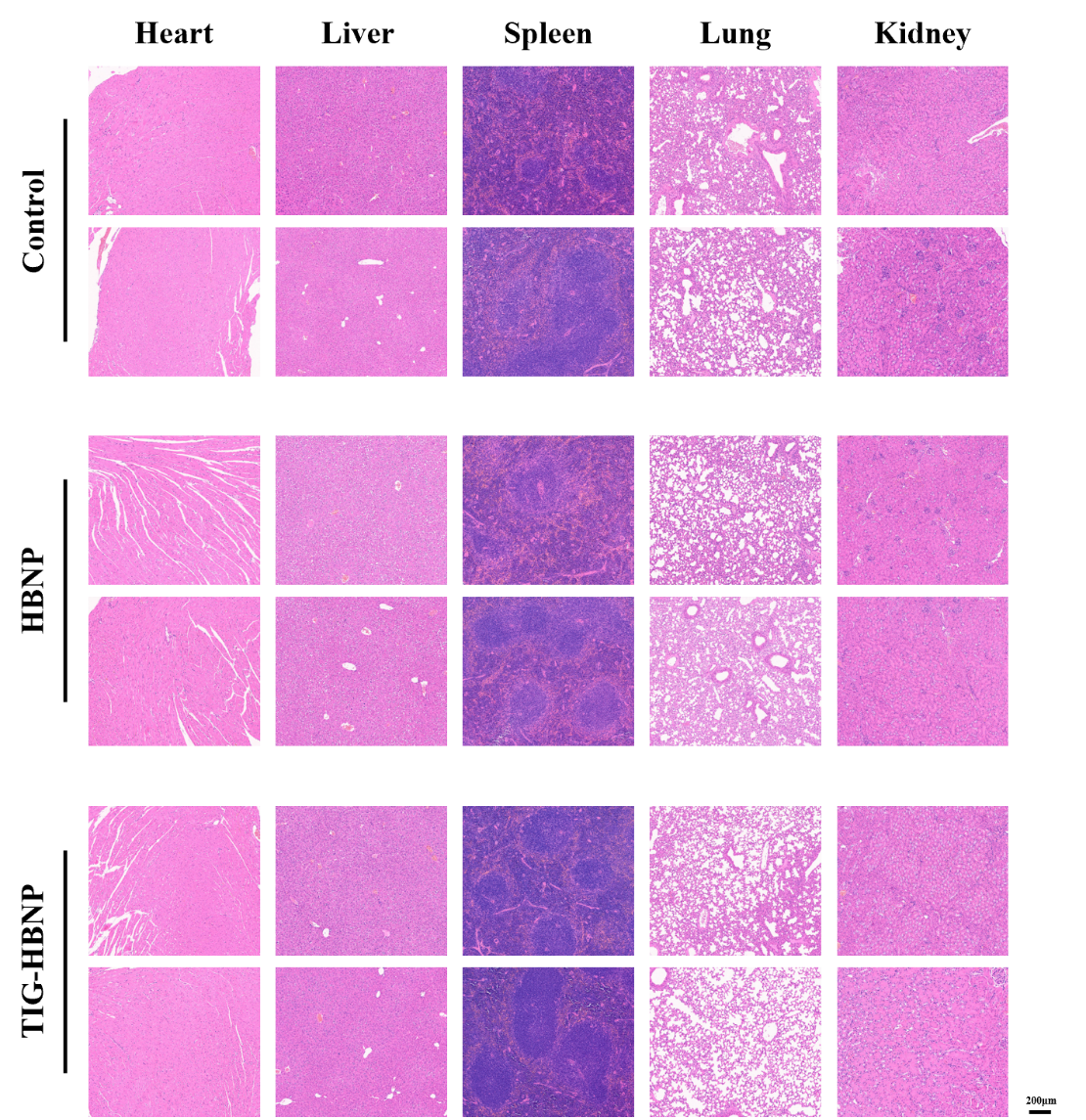


Fig. S8 HE staining of heart, liver, spleen, lung, and kidney. Scale bar: 200 μm —— That is: Supplementary to Fig. 6D.
