# Supplementary figures and images for: A Hemoglobin-Based Nanoparticle Delivery System Enhances the Pharmacokinetics and Efficacy of Tigecycline in Klebsiella pneumoniae Infections
Source: BME Front. 2026 Mar 30;7:0241. doi: 10.34133/bmef.0241 (PMC13033835; doi:10.34133/bmef.0241)

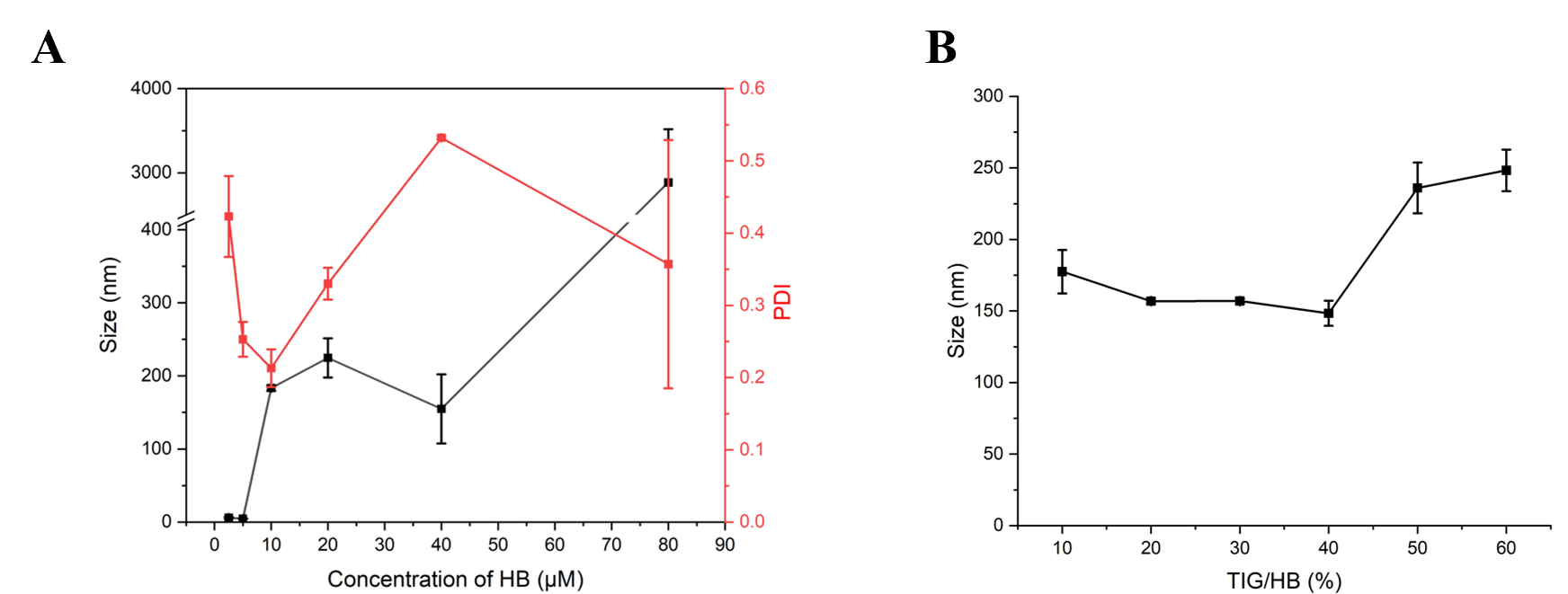

Supplement: Supplementary 1 — Graphical Abstract Figs. S1 to S8 [file bmef.0241.f1.zip › Figure S1.png]

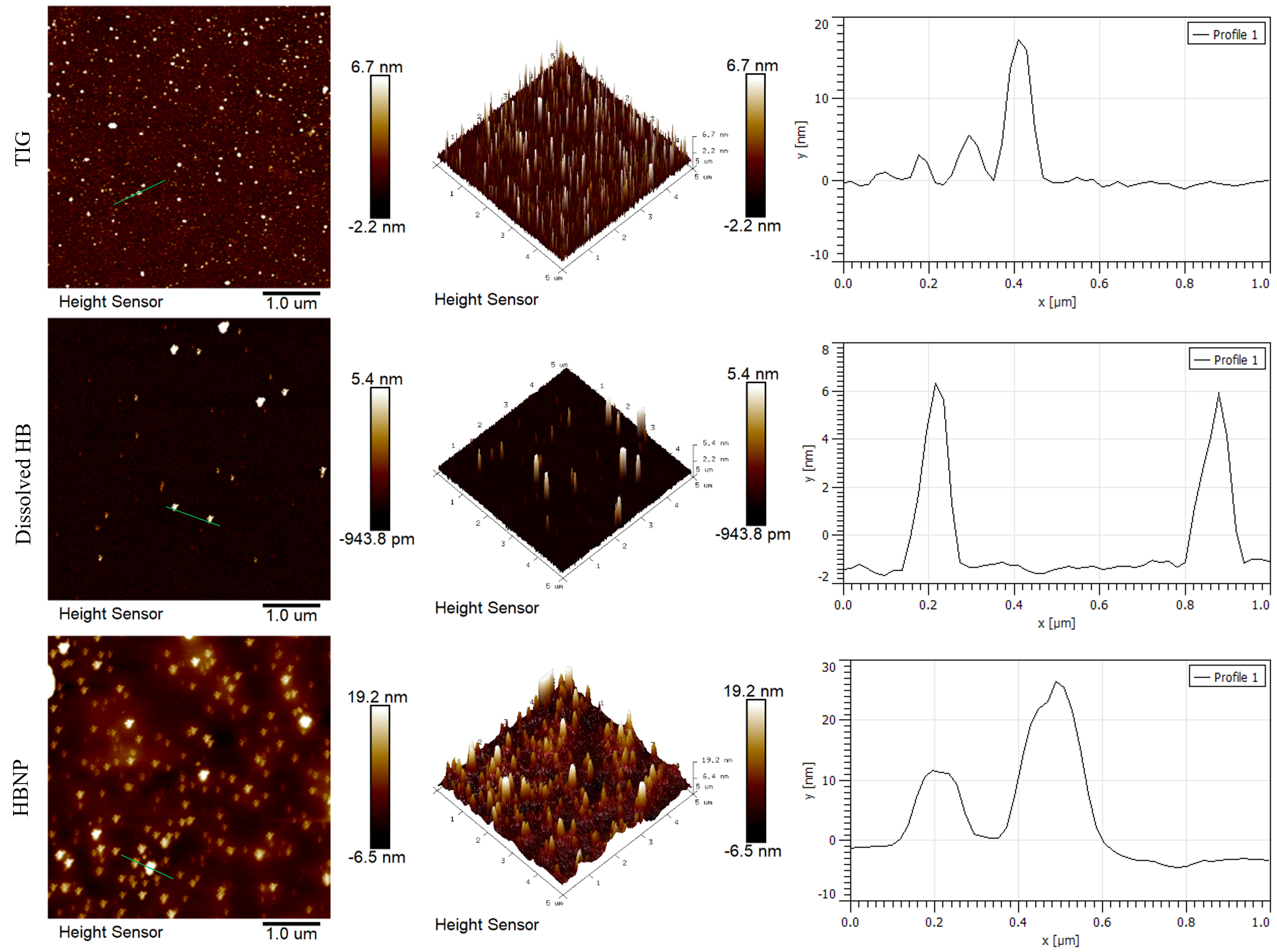

Supplement: Supplementary 1 — Graphical Abstract Figs. S1 to S8 [file bmef.0241.f1.zip › Figure S2.png]

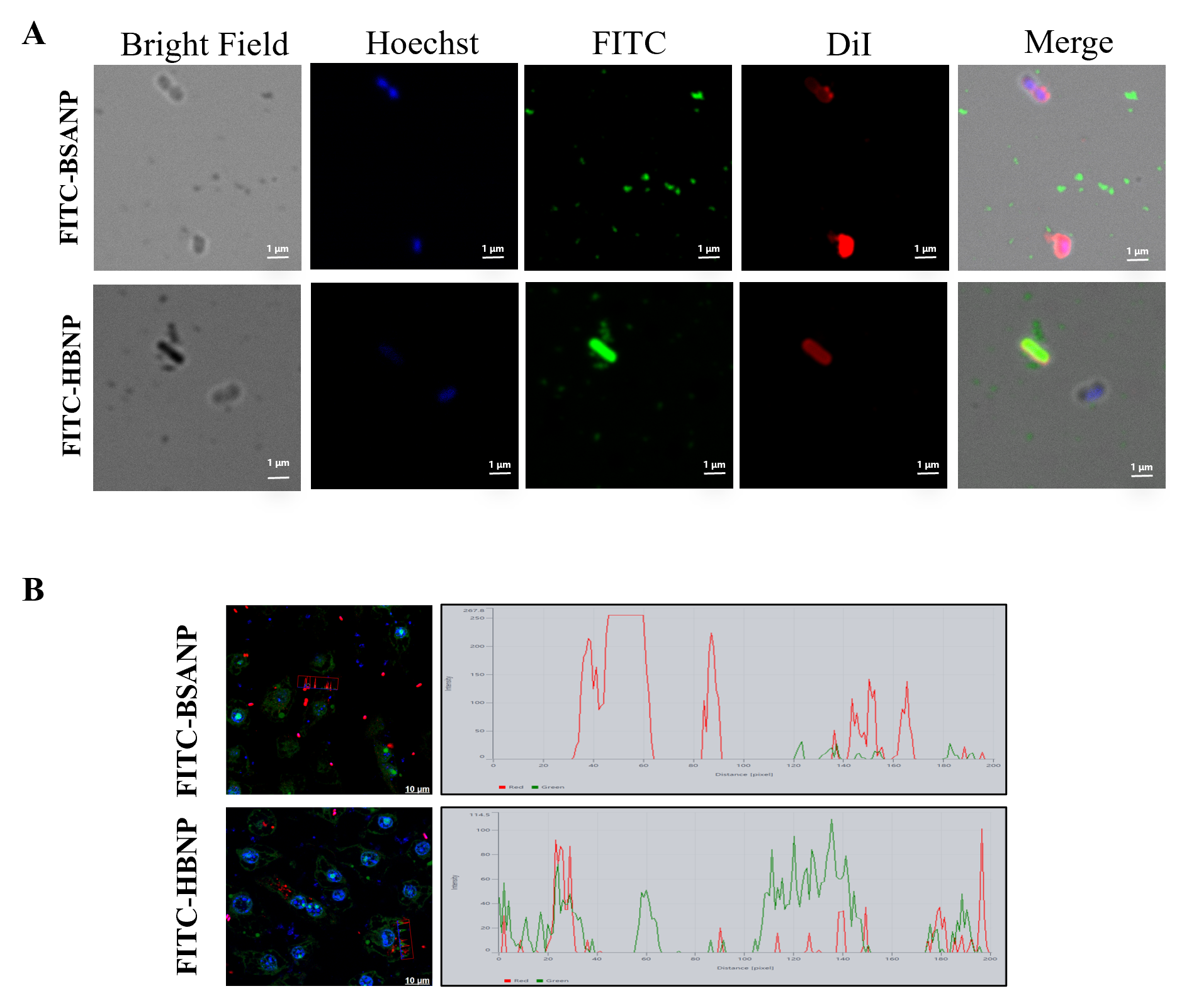

Supplement: Supplementary 1 — Graphical Abstract Figs. S1 to S8 [file bmef.0241.f1.zip › Figure S3.png]

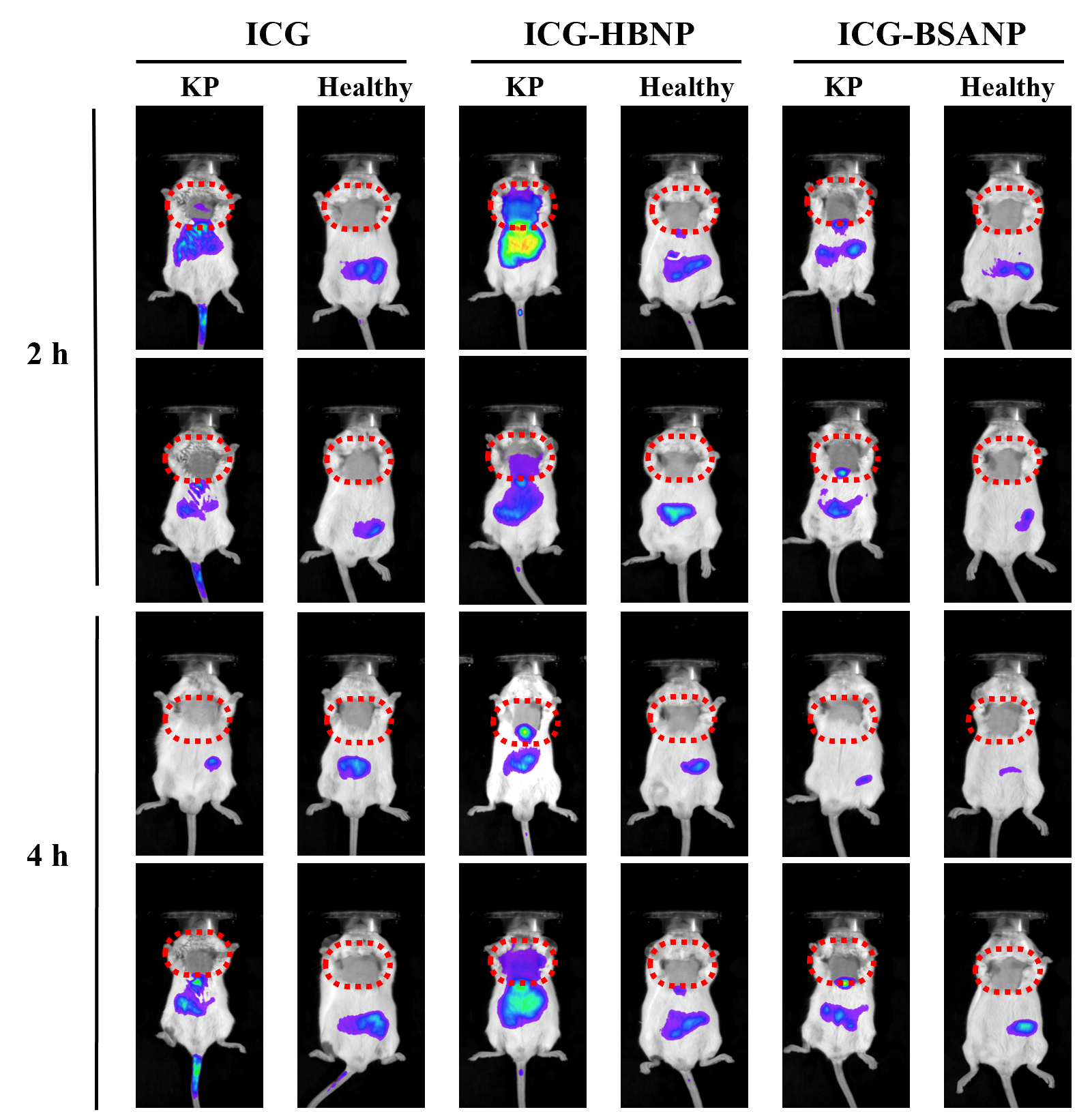

Supplement: Supplementary 1 — Graphical Abstract Figs. S1 to S8 [file bmef.0241.f1.zip › Figure S4.png]

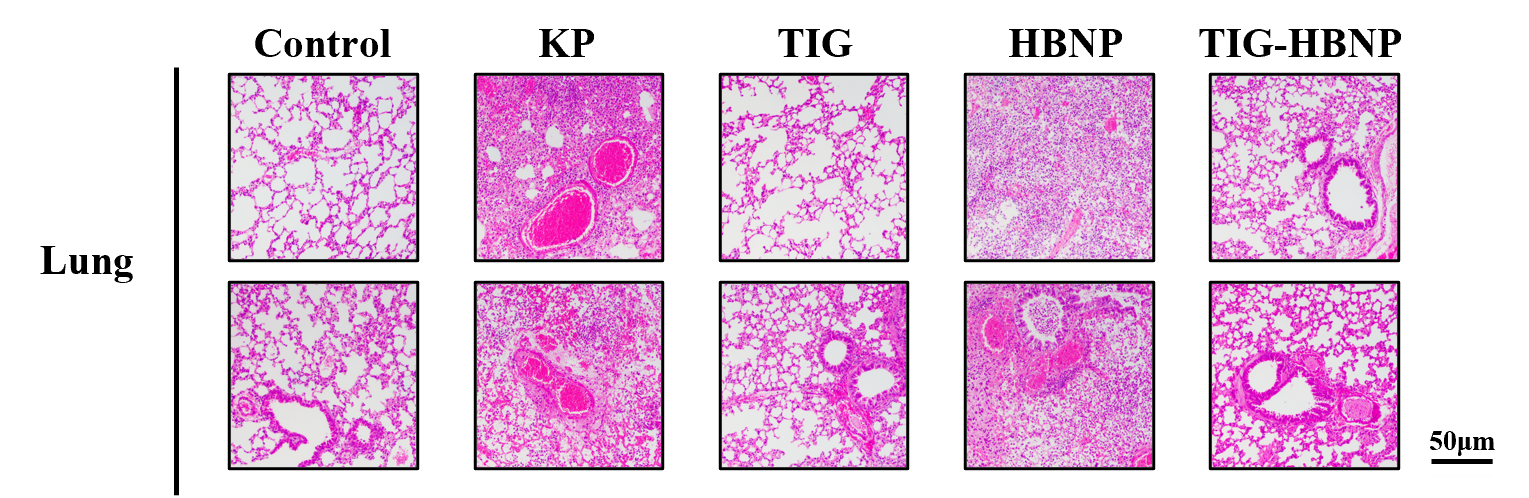

Supplement: Supplementary 1 — Graphical Abstract Figs. S1 to S8 [file bmef.0241.f1.zip › Figure S5.png]

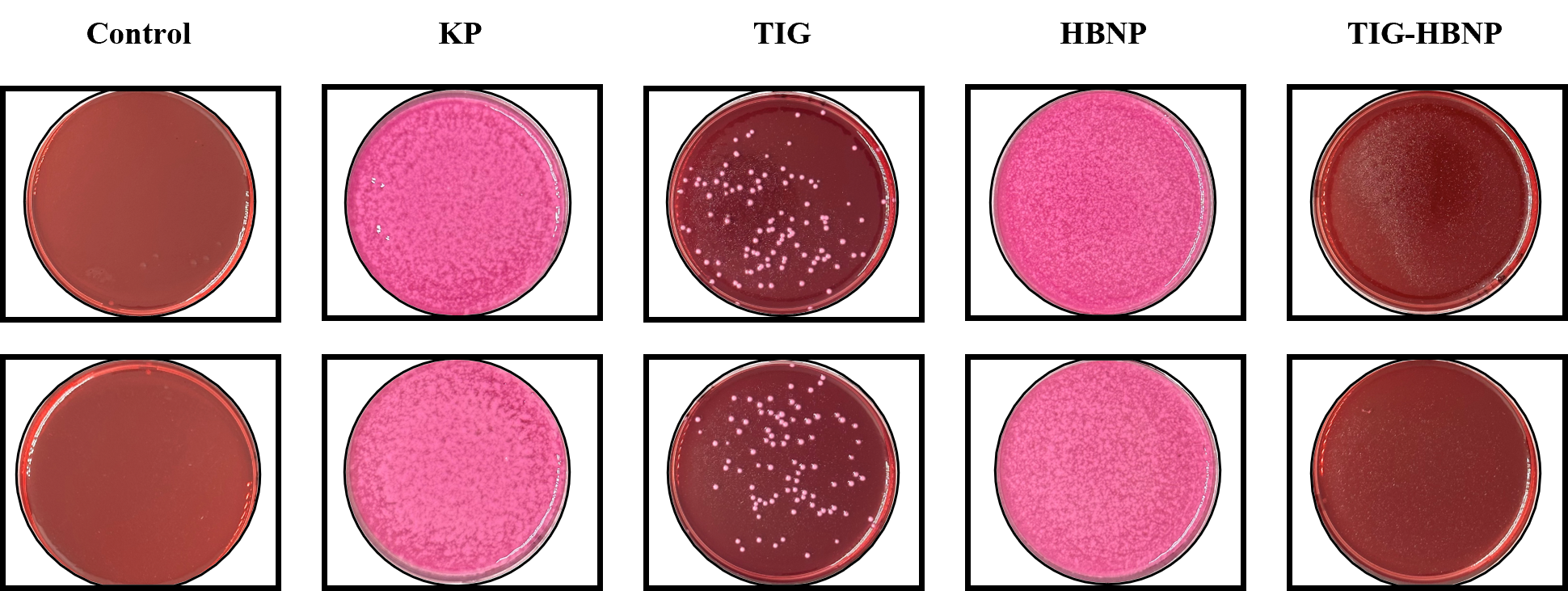

Supplement: Supplementary 1 — Graphical Abstract Figs. S1 to S8 [file bmef.0241.f1.zip › Figure S6.png]

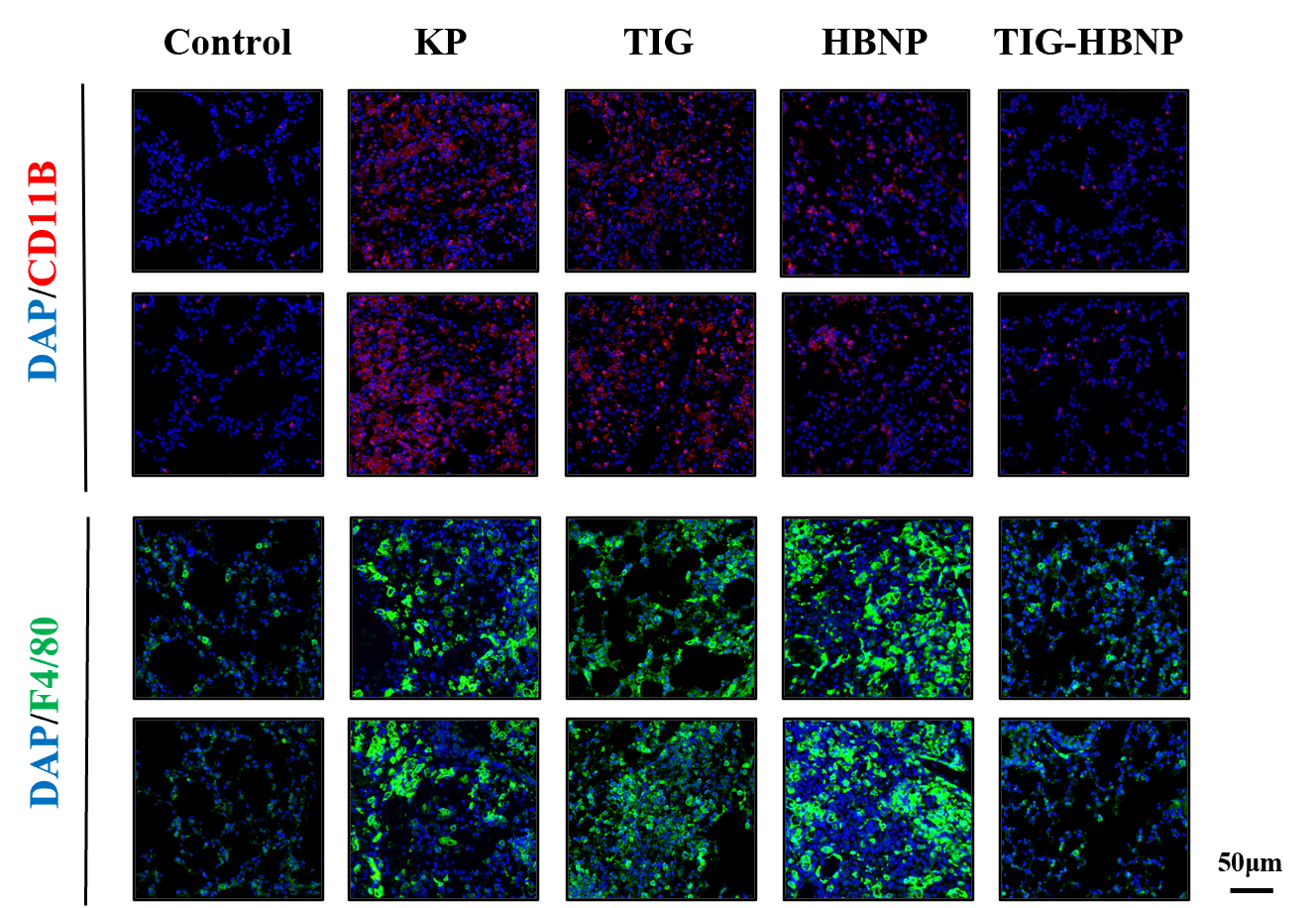

Supplement: Supplementary 1 — Graphical Abstract Figs. S1 to S8 [file bmef.0241.f1.zip › Figure S7.png]

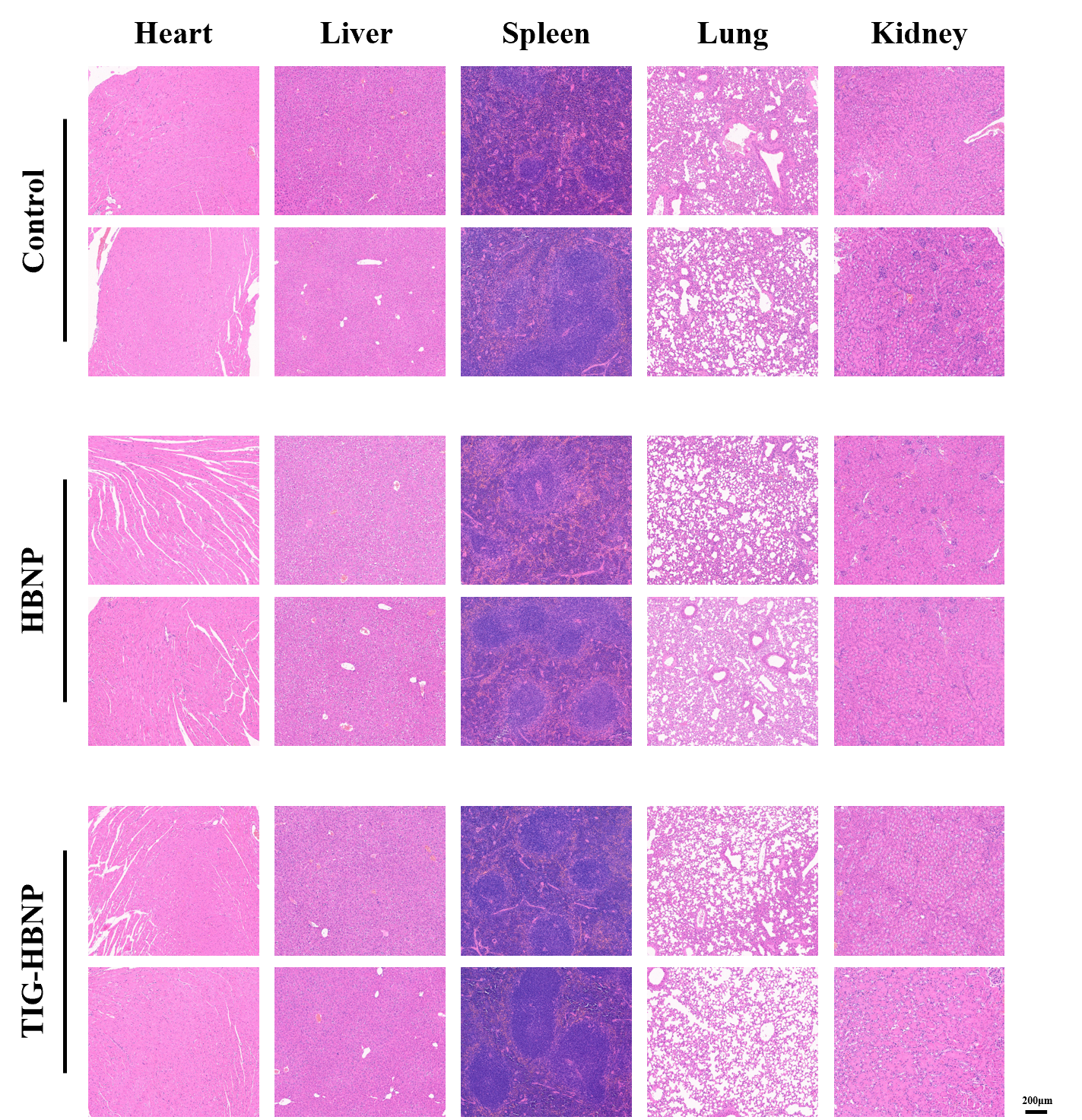

Supplement: Supplementary 1 — Graphical Abstract Figs. S1 to S8 [file bmef.0241.f1.zip › Figure S8.png]

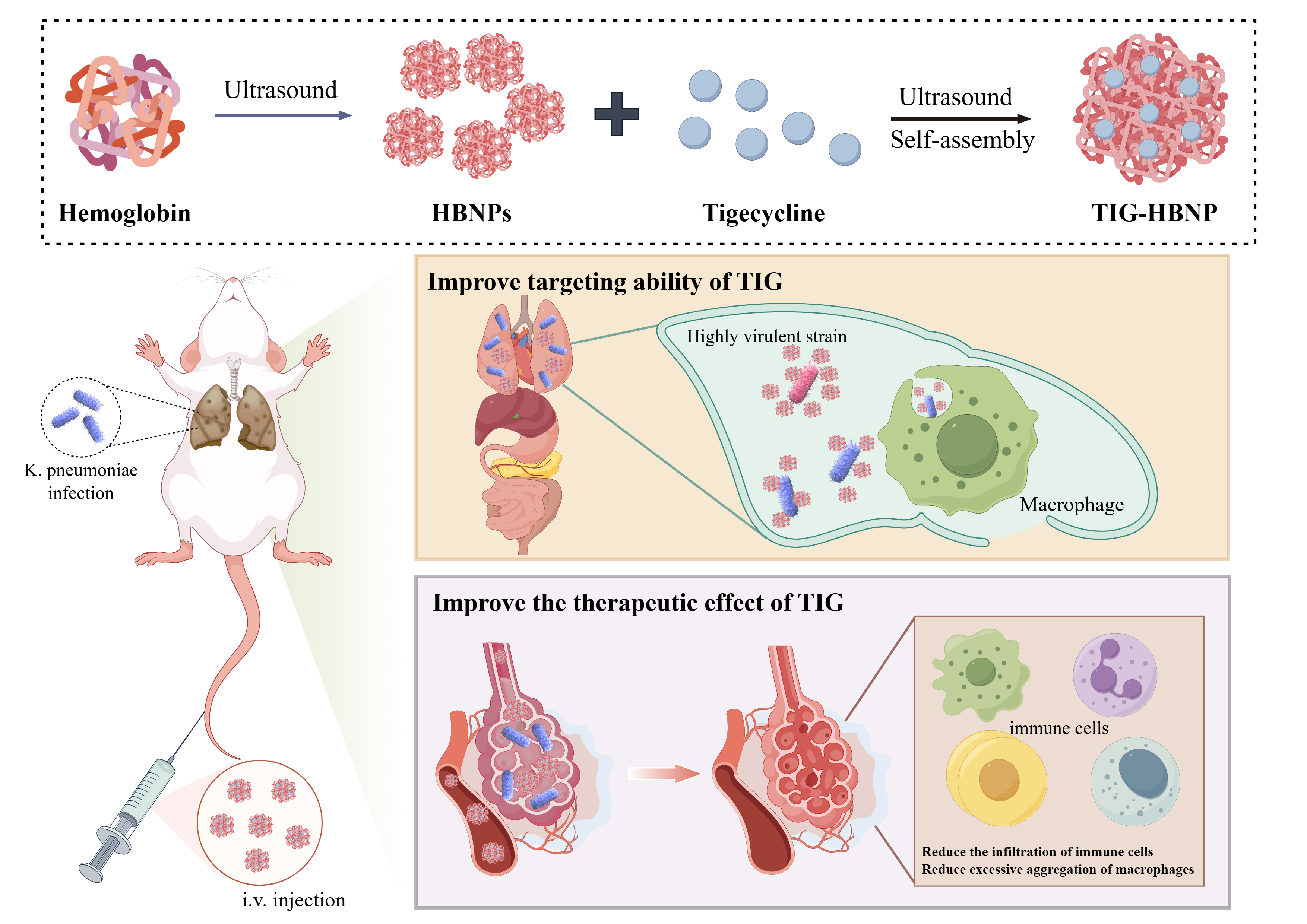

Supplement: Supplementary 1 — Graphical Abstract Figs. S1 to S8 [file bmef.0241.f1.zip › Graphical abstract.tiff]
